# Foxp1 and Lhx1 Coordinate Motor Neuron Migration with Axon Trajectory Choice by Gating Reelin Signalling

**DOI:** 10.1371/journal.pbio.1000446

**Published:** 2010-08-10

**Authors:** Elena Palmesino, David L. Rousso, Tzu-Jen Kao, Avihu Klar, Ed Laufer, Osamu Uemura, Hitoshi Okamoto, Bennett G. Novitch, Artur Kania

**Affiliations:** 1 Neural Circuit Development Laboratory, Institut de recherches cliniques de Montréal (IRCM), Montréal, Canada; 2 Department of Neurobiology, Broad Center of Regenerative Medicine and Stem Cell Research, David Geffen School of Medicine at UCLA, Los Angeles, California, United States of America; 3 Department of Medical Neurobiology, Institute for Medical Research-Israel-Canada (IMRIC), Hebrew University-Hadassah Medical School, Jerusalem, Israel; 4 Department of Genetics and Development, Columbia University, New York, New York, United States of America; 5 National Hospital Organization Murayama Medical Center, Tokyo, Japan; 6 Laboratory for Developmental Gene Regulation, RIKEN Brain Science Institute, Saitama, Japan; 7 Faculté de Médecine, Université de Montréal, and Departements of Anatomy and Cell Biology and Biology, Division of Experimental Medicine, McGill University, Montréal, Canada; University North Carolina, United States of America

## Abstract

Topographic neuronal maps arise as a consequence of axon trajectory choice correlated with the localisation of neuronal soma, but the identity of the pathways coordinating these processes is unknown. We addressed this question in the context of the myotopic map formed by limb muscles innervated by spinal lateral motor column (LMC) motor axons where the Eph receptor signals specifying growth cone trajectory are restricted by Foxp1 and Lhx1 transcription factors. We show that the localisation of LMC neuron cell bodies can be dissociated from axon trajectory choice by either the loss or gain of function of the Reelin signalling pathway. The response of LMC motor neurons to Reelin is gated by Foxp1- and Lhx1-mediated regulation of expression of the critical Reelin signalling intermediate Dab1. Together, these observations point to identical transcription factors that control motor axon guidance and soma migration and reveal the molecular hierarchy of myotopic organisation.

## Abstract 2

During embryonic development of the vertebrate motor system, the same transcription factors that regulate axonal trajectories can also regulate cell body migration, thereby controlling topographic map formation.

## Author Summary

Many areas of our nervous system are organized in a topographic manner, such that the location of a neuron relative to its neighbors is often spatially correlated with its axonal trajectory and therefore target identity. In this study, we focus on the spinal myotopic map, which is characterized by the stereotyped organization of motor neuron cell bodies that is correlated with the trajectory of their axons to limb muscles. An open question for how this map forms is the identity of the molecules that coordinate the expression of effectors of neuronal migration and axonal guidance. Here, we first show that Dab1, a key protein that relays signals directing neuronal migration, is expressed at different concentrations in specific populations of limb-innervating motor neurons and determines the position of their cell bodies in the spinal cord. We then demonstrate that Foxp1 and Lhx1, the same transcription factors that regulate the expression of receptors for motor axon guidance signals, also modulate Dab1 expression. The significance of our findings is that we identify a molecular hierarchy linking effectors of both neuronal migration and axonal projections, and therefore coordinating neuronal soma position with choice of axon trajectory. In general, our findings provide a framework in which to address the general question of how the nervous system is organized.

## Introduction

Neural circuits are frequently organised in a topographic manner such that the position of a neuronal cell body is correlated with the location of the post-synaptic target and therefore its axon trajectory. Since the inference of such organisational principles [1], the molecular identity of many neuronal migration and axon guidance cues has been uncovered [2],[3]. Recent studies have also begun to identify the transcription factors that control neuronal identity and deploy the repertoire of neuronal migration and axon guidance receptors and signals employed in neural circuit assembly [4],[5],[6]. These observations raise the possibility that correlated neuronal soma localisation and axon trajectory of topographically ordered neural circuits arise as a consequence of specific transcription factors directing both axon guidance and cell body migration effector expression.

Vertebrate spinal motor neurons are organised myotopically in longitudinal columns such that the location of their soma in the ventral spinal cord corresponds to the position of their muscle targets in the periphery [7]. In mouse and chick, motor neurons innervating axial and body wall muscles are located in medially positioned columns, whereas motor neurons innervating limb muscles are located in the lateral motor column (LMC) present only at spinal cord levels in register with limbs. LMC neurons are further subdivided according to their axon trajectory within the limb: lateral LMC (LMCl) neurons innervate dorsal limb muscles, whereas medial LMC (LMCm) neurons innervate ventral limb muscles [8],[9],[10]. Motor pools are also organised myotopically such that, in general, the anterio-posterior location of a pool within the LMC correlates with the proximo-distal location of its limb muscle target [7],[9],[11],[12]. A motor axon guidance decision point is at the base of the limb where LMC axons interact with mesenchymal cells resulting in the selection of a dorsal or a ventral limb nerve trajectory [10],[13]. Concomitant with this process, LMC somata migrate from the progenitor-rich ventricular zone to the ventral horn of the spinal cord [14],[15], with the later-born LMCl neurons migrating past the earlier-born LMCm neurons in a manner reminiscent of the inside-out lamination of the developing cerebral cortex [16],[17],[18]. Recent studies also describe a topographic relationship between motor neuron soma and dendrite localisation in *Drosophila* and the patterns of motor neuron recruitment during swimming in fish [19],[20].

The molecular signals controlling the trajectory of LMC axons are characterised, but those controlling LMC soma position in the spinal cord are poorly understood. The LIM homeodomain proteins Isl1 and Lhx1, expressed by LMCm and LMCl neurons respectively, act in conjunction with the pan-LMC forkhead domain transcription factor Foxp1 to specify the dorsoventral axon trajectory in the limb by regulating the expression of axonal Eph tyrosine kinase receptors that enable LMC growth cones to respond to ephrin ligands in the limb mesenchyme. Genetic evidence argues that ephrin-A ligands in the ventral limb repulse EphA-expressing LMCl axons into the dorsal limb nerve, while ephrin-B ligands in the dorsal limb repulse EphB-expressing LMCm axons into the ventral limb nerve [21],[22],[23],[24],[25],[26]. The clustering of some motor pools relies on EphA4, type II cadherins, and the ETS transcription factor Pea3 [27],[28],[29], while migration of LMCl and LMCm neurons into their appropriate columnar location can be biased by Lhx1 and Isl1 and requires Foxp1 [21],[22],[23]. These observations raise the possibility that Foxp1, Lhx1, and Isl1 control the migration of LMC cell bodies within the ventral horn by restricting the expression of specific effectors of neuronal migration.

The extracellular matrix protein Reelin is a crucial neuronal migration signal that acts through the lipoprotein receptors VLDLR or ApoER2 to induce the phosphorylation of the intracellular adaptor protein Dab1 leading to remodelling of the actin cytoskeleton [30]. Loss of Reelin or its signalling effectors disrupts the layering of the neuronal somata within the cerebral cortex [31],[32],[33] but the role of Reelin in neuronal migration remains controversial. Reelin has been proposed to act as a neuronal migration stop signal [34]; however, since Reelin expression in the ventricular zone can partially rescue the pre-plate splitting defects in Reelin-deficient mice, Reelin has also been proposed to act as a permissive signal enabling neurons to interpret distinct migration cues [35]. Similar to cortical neurons, spinal neuron progenitor clones migrate away from the ventricular zone in radial spoke-like trajectories [14] and the migration of preganglionic (PG) motor neurons and the layering of the dorsal horn laminae is controlled by Reelin [36],[37]. These studies raise the possibility that Reelin may also regulate the localisation of LMC neurons and is thus a general migration cue specifying the position of many different classes of spinal neurons including LMC motor neurons.

Using gain and loss of function experiments in chick and mouse, we provide evidence that Reelin directs LMC neuron migration but not the selection of limb axon trajectory. We also show that Foxp1 and Lhx1, the transcription factors specifying LMC axon trajectory choice, gate Reelin signalling through the restriction of Dab1, a key signalling intermediate. Thus, the same transcription factors are directing neuronal soma migration and axon trajectory selection revealing the molecular hierarchy controlling the establishment of a somatotopic map.

## Results

### Expression of Reelin, VLDLR, ApoER2, and Dab1 in the Ventral Spinal Cord

To explore the possibility that Reelin signalling might control LMC soma migration, we monitored the expression of Reelin, its receptors, and their adaptor protein Dab1 in mouse embryos between embryonic day of development (e) 11.5 and e12.5 and in chick embryos between Hamburger and Hamilton (HH) stages (St) 23 and 30 [38] in limb-level spinal cord. These stages correspond to the times at which LMCl neurons are migrating out of the ventricular zone and reach their final position lateral to LMCm neurons [17],[22]. We used the transcription factor Foxp1 as a pan-LMC marker and subdivided the LMC based on the presence of Isl1 and Lhx1 transcription factors [21],[23],[25].

**Figure 1:**
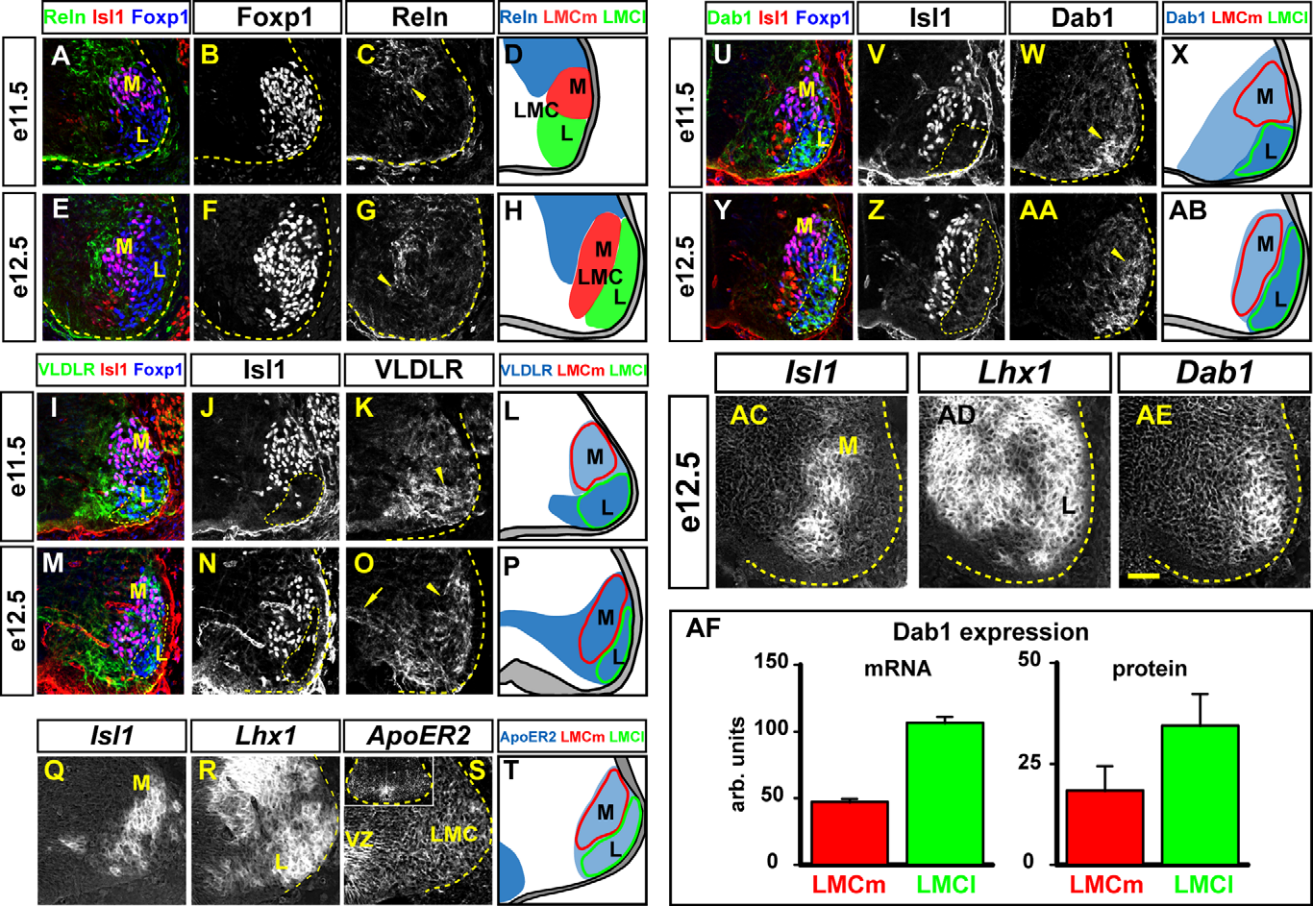
Ventral spinal cord expression of Reelin, VLDLR, *ApoER2* and Dab1. (A–P, U–AB) Protein detection in e11.5 and e12.5 mouse lumbar spinal cord. LMCm (M) and LMCl (L) neurons are identified as Foxp1^+^ Isl1^+^ and Foxp1^+^ Isl1^−^, respectively. Reelin is expressed in a domain medio-dorsal to the LMC at e11.5 (A–C) and expands more ventrally at e12.5 (G; arrowheads). VLDLR expression is stronger in LMCl than in LMCm neurons (arrowheads) at e11.5 (I–K). Arrow in (O) indicates VLDLR protein in neuronal processes extending towards the ventricular zone. Dab1 expression is stronger in LMCl neurons (arrowheads in W and AA) than in LMCm neurons at both e11.5 (U–W) and e12.5 (Y–AA). (Q–S, AC–AE) Detection of mRNA in consecutive lumbar spinal cord sections of an e11.5 (Q–S) and e12.5 (AC–AE) mouse embryo. *Isl1* (Q, AC) and *Lhx1* (R, AD) expression highlights LMCm and LMCl motor neurons, respectively. *ApoER2* mRNA is detected throughout the ventral spinal cord (S), with higher levels of expression in the ventricular zone (VZ). Inset in (S) shows a lower magnification image of the same section, highlighting the specificity of the probe. *Dab1* mRNA expression levels are higher in LMCl neurons than in LMCm neurons (AE). (AF) Quantifications of *Dab1* mRNA and protein levels in LMCm (Isl1^+^ Foxp1^+^) and LMCl (Isl1^−^ Foxp1^+^) in lumbar spinal cord of e12.5 mouse embryos. Quantifications were gated on Lhx1^+^ LMCl and Isl1^+^ LMCm regions in neighbouring sections and represent a mean intensity value within the gated region with the background (gating on the ventricular zone) subtracted. arb: arbitrary units on a 0 (no signal) to 255 (maximum intensity) scale. Measurements are based on three embryos and six sections. (D, H, L, P, T, X, AB) Schematic representation of LMCm, LMCl, and Reelin signalling components. Blue intensity varies with expression levels. Stippled lines outline the spinal gray matter and LMCl neurons. Scale bar: 65 µm (A–AB), 50 µm (AC–AE).

Reelin has previously been detected in the thoracic spinal cord adjacent to PG neurons [36]. At limb levels Reelin is expressed from e10.5 (Figure S1) and in e11.5 mouse embryos we observed Reelin expression in cells medio-dorsal to LMC neurons, and by e12.5 this domain expanded ventrally, resulting in a Reelin-rich band intercalated between the ventricular zone and the LMC (Figure 1A–H). We also observed a similar *Reelin* mRNA and protein distribution in chick embryos (Figure S1).

We next monitored the expression of Reelin receptors VLDLR and ApoER2 and their intracellular adaptor protein Dab1 in mouse and chick spinal cords. In e11.5 mouse embryos at both limb levels, VLDLR protein and mRNA were apparently expressed in all LMC neurons (Figure 1I–L; unpublished data). However, VLDLR protein levels appeared higher in LMCl neurons relative to LMCm neurons (Figure 1K). By e12.5 *VLDLR* mRNA and protein levels appeared uniform throughout the LMC (Figure 1M–P; unpublished data). In chick embryos, *VLDLR* mRNA was present in apparently all lumbar LMC neurons at both HH St 24 and HH St 30 (Figure S1). At the stages examined, *ApoER2* mRNA was expressed in the ventricular zone adjacent to the floor plate of both mouse and chick embryos; however, its expression in LMC neurons was only apparent in mouse embryos (Figure 1Q–T; Figure S1; unpublished data).

In mouse, *Dab1* mRNA and protein were present throughout the LMC from e10.5, at both limb levels; however, at later ages examined, an LMC subpopulation expressed *Dab1* mRNA and protein at noticeably higher levels (Figure 1U–AF; Figure S1, Figure S4; unpublished data). At e11.5, this expression domain (Dab1^high^) was confined to the medio-ventral aspect of the LMC corresponding to Foxp1^+^Isl1^−^ LMCl neurons while the low-level Dab1 expression domain (Dab1^low^) was confined to the dorsally positioned Isl1^+^Foxp1^+^ LMCm neurons (Figure 1U–X). By e12.5, Dab1^high^ and Dab1^low^ LMC neurons were found in, respectively, lateral and medial aspect of the LMC, and corresponded to LMCl and LMCm neurons (Figure 1Y–AB). Similar *Dab1* mRNA distribution was observed in chick embryos (Figure S1). Together, our expression data raise the possibility that Reelin signalling directs LMC soma migration and the disparate Dab1 expression levels in LMCl and LMCm neurons suggest that these neuronal populations may differ in their responsiveness to Reelin.

### LMC Migration Defects in *Dab1* and *Reln* Mutant Mice

**Figure 2:**
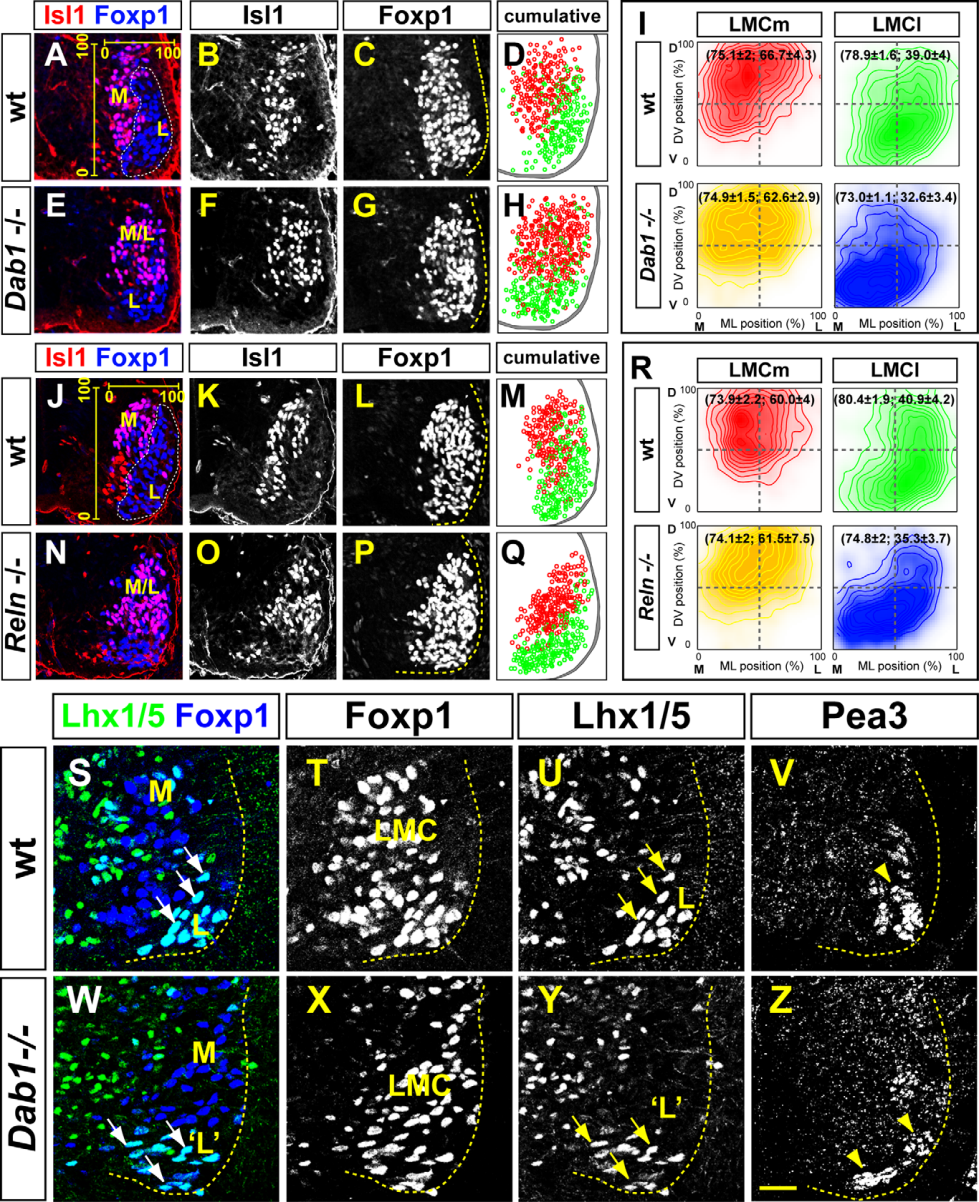
Impaired LMC position in *Dab1* and *Reln* mutants. (A–H, J–Q) LMCm (Foxp1^+^ Isl1^+^) and LMCl (Foxp1^+^ Isl1^−^) neurons in the lumbar spinal cord of e12.5 *Dab1* mutants (E–H), *Reln* mutants (N–Q), and wild type littermates (A–D, J–M). In both mutants, LMCl neurons are positioned more ventrally, while LMCm neurons are shifted laterally, relative to control embryos. Some overlap between LMCl and LMCm domain is also evident (M/L in panels E and N). Superimposed ventral spinal cord position (D, H, M, Q) of LMCm (red) and LMCl (green) neurons in several consecutive sections of representative embryos highlights the altered position of LMC neurons. (I, R) Density plots of mediolateral (ML) and dorsoventral (DV) position of LMCm and LMCl neurons as percentage of LMC width and height. See Materials and Methods for details. Mean position (ML, DV) is in brackets. (I) Hotelling's T^2^ test *p* values: LMCm(wt) versus LMCm(*Dab1*), *p* = 0.2925; LMCl(wt) versus LMCl(*Dab1*), *p* = 0.0035. *n* = 4 (*Dab1*) and 6 (wt) embryos; *N*>1,500 neurons per genotype. (R) Hotelling's T^2^ test *p* values: LMCm(wt) versus LMCm(*Reln*), *p* = 0.9024; LMCl(wt) versus LMCl(*Reln*), *p* = 0.0473. *n* = 4 embryos per genotype; *N*>1,500 neurons per genotype. (S–Z) Detection of Foxp1, Lhx1/5, and Pea3 on consecutive sections of lumbar spinal cord of e15.5 *Dab1* mutants (W–Z) and control littermate (S–V). In *Dab1* mutants, LMCl (Foxp1^+^ Lhx1/5^+^) neurons are positioned medio-ventrally relative to control embryos. The position and clustering of the Pea3^+^ (Isl1^−^ Foxp1^+^) motor pool in the e15.5 lumbar spinal cord of *Dab1* mutants (Z) is disrupted compared to control embryos (V). All values are expressed as mean ± s.d. Dashed lines divide the plots into four equal quadrants. Stippled lines outline the spinal grey and LMCl neurons. Scale bar: 77 µm (A–H, J–Q), 50 µm (S–Z).

To determine whether Reelin signalling influences LMC neuron migration, we examined the spinal cord of *Dab1* and *Reelin* (*Reln*) mutant mice (Figure 2) [31],[32]. Since Reelin signalling is required for the appropriate positioning of PG neurons which share a part of their migration trajectory with LMC neurons [36],[39], we focused our analysis on caudal lumbar-sacral (LS) levels, which contain no PG neurons, as assessed by phospho-Smad1 expression [23]. During LMC migration, the total number of LMC neurons, LMCl and LMCm subtype specification, and radial glia development was unaffected by *Dab1* and *Reln* loss of function (Figure S2, Figure S3; unpublished data). Additionally, most likely because of its impaired degradation [40], Dab1 protein levels in LMC neurons were increased in *Reln* mutants, suggesting that all LMC neurons are responsive to Reelin (Figure S4).

We next analysed the localisation of lumbar LMC neurons in *Dab1* and *Reln* mutants at e12.5, the time at which, in control embryos, the majority of wild type LMCl neurons have terminated their migration and are positioned lateral to LMCm neurons (Figure 2A–D). In *Dab1* mutants, LMCl neurons settled ventral to LMCm neurons, which were abnormally shifted to a lateral position in the ventral horn, and many LMCl and LMCm neurons were intermingled (Figure 2E–H). This neuronal displacement was more evident when we superimposed the position of LMCl and LMCm neurons in images of adjacent wild type (wt) and *Dab1* mutant spinal cords sections (Figure 2D, H). To assess the expressivity of this phenotype and to account for LMC neuron displacement along mediolateral (ML) and dorsoventral (DV) axes simultaneously, we performed a two-dimensional position analysis of LMC neuron position using the bivariate statistical Hotelling's T^2^ test. We measured the mean ML and DV coordinates of wild type and *Dab1* mutant LMC neurons within the ventral spinal cord. To compensate for sectioning artefacts, we normalised the ML coordinates to the distance from the ventricular zone to the lateral edge of the Foxp1^+^ expression domain and the DV coordinates to the dorsoventral extent of the Foxp1^+^ expression domain, two standard measurements that are not different between *Dab1* mutants and wild type littermates (see Experimental procedures for details; unpublished data). Thus, with the lateral-most edge of the LMC defined as ML: 100%, and with the dorsal-most domain of the LMC defined as DV: 100%, in wild type embryos, the mean position of LMCm neurons was not changed significantly by *Dab1* mutation; however, these neurons were spread over a larger mediolateral zone compared to wild type littermates (Figure 2I; Table S2). In contrast, by visual inspection of at least six spinal cord sections per embryo, we noted that in six out of six embryos analysed, LMCl neurons were positioned aberrantly. Quantification revealed that LMCl neuron position was significantly shifted in a medio-ventral direction in *Dab1* mutants relative to wild type littermates ((ML: 73%; DV: 33%) versus (ML: 79%; DV: 39%); *p*<0.0035, Hotelling's T^2^ test; Table S2), which could be observed at least until e15.5 (Figure 2S–U, W–Y; unpublished data). A similar LMC migration phenotype was also observed in the cervical spinal cord as well (unpublished data), and in chick LMC neurons expressing a Dab1 protein in which the five tyrosines essential for Reelin signalling have been mutated (Dab1^5YF^; Figure S5, Table S3; [41]). We also noted that in four out of four embryos, the position within the ventral spinal cord of a Pea3-expressing motor neuron pool was shifted medio-ventrally at e15.5 (Figure 2V, Z). Together, these results demonstrate that in the limb-level spinal cord, Dab1 is essential for the normal migration of LMC neurons and motor pool position.

We next examined the position of lumbar LMC neurons in *Reln* mutant embryos at e12.5: *Reln* mutation did not alter the mean position of LMCm neurons (Figure 2J–Q; Table S2), although as in *Dab1* ^−/−^ embryos, these neurons were spread over a larger area of the LMC when compared to controls (Figure 2R). In contrast, in three out of four embryos, we observed that LMCl neurons were positioned abnormally, with quantification revealing that the mean LMCl neuron position in *Reln* mutants was significantly shifted in the medio-ventral direction relative to wild type, with many LMCl neurons found intermingled with LMCm neurons ((ML: 75%; DV: 35%) versus (ML: 80%; DV: 41%); *p*<0.0473, Hotelling's T^2^ test; Figure 2J–R; Table S2). Migration defects observed in *Reln* mutants mirrored those observed in *Dab1* mutants, thus implicating Reelin signalling in the specification of LMC soma position in the ventral spinal cord.

### Dab1 Expression Determines LMC Soma Position

**Figure 3:**
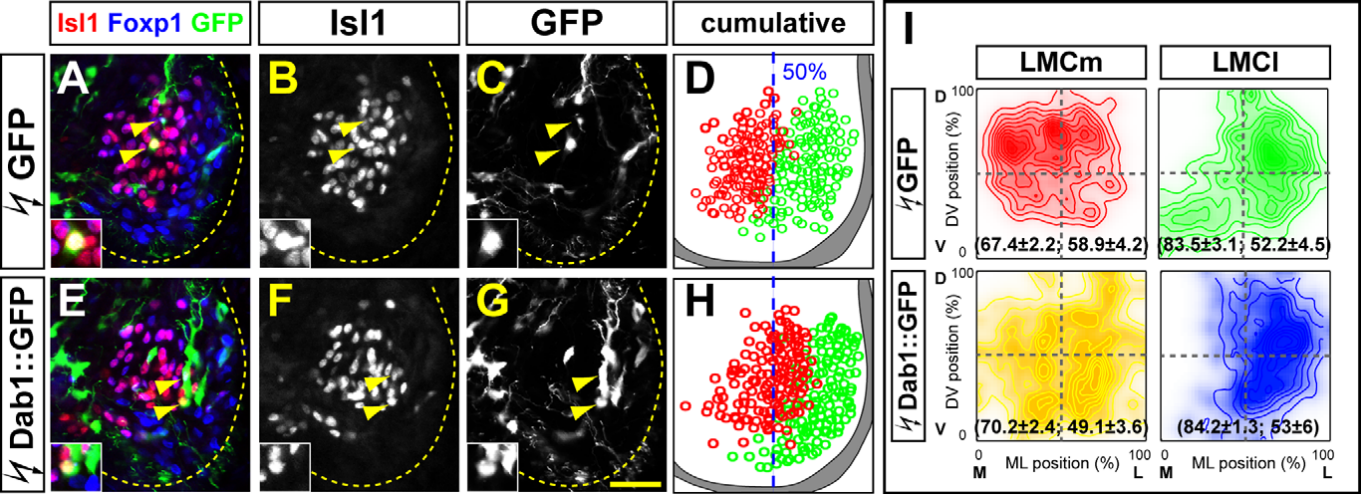
Dab1 is sufficient to specify LMC neuron position. (A–C, E–G) GFP expression in LMCm (Foxp1^+^Isl1^+^) and LMCl (Foxp1^+^Isl1^−^) neurons in lumbar spinal cord of chick HH St 29 embryos electroporated with GFP (A–C) or Dab1::GFP expression plasmids (E–G). Arrowheads point to electroporated LMCm neurons shown in insets at higher magnification. (D, H) Superimposed ventral spinal cord location of electroporated LMCm (red) and LMCl (green) neurons in several consecutive sections of representative embryos highlighting the laterally shifted position of Dab1-overexpressing LMCm neurons. Blue dashed line indicates the 50% ML value. (I) Density plots of mediolateral (ML) and dorsoventral (DV) position of LMCm and LMCl neurons as percentage of LMC width and height. Mean position (ML, DV) is indicated in brackets. Hotelling's T^2^ test *p* values: LMCm(GFP) versus LMCm(Dab1::GFP), *p* = 0.0165; LMCl(GFP) versus LMCl(Dab1::GFP), *p* = 0.9019. *n* = 4 (GFP), 5 (Dab1::GFP) embryos; *N*>60 neurons per embryo per experimental condition. Dashed lines divide LMC in four equal quadrants. All values are expressed as mean ± s.d.. Stippled lines outline the spinal gray matter. Scale bar: 20 µm.

Based on the differential expression and the requirement for its function in LMCm and LMCl neurons, we reasoned that the levels of Dab1 expression, rather than simply its presence or absence, might influence the migration of LMC neurons. We therefore asked whether increasing Dab1 expression would shift the position of LMC soma laterally. To do this, we used *in ovo* electroporation to introduce a Dab1::GFP fusion protein or GFP expression plasmids into the lumbar spinal cord of HH St 17/19 embryos and monitored the position of GFP^+^ LMC neurons at HH St 29 [22]. Dab1::GFP was expressed with equal efficiency in LMCl and LMCm neurons and did not change their identity nor affect their axon trajectory in the limb (Figure S6; unpublished data). The mean position of LMCl neurons with elevated Dab1 levels was the same as that of LMCl neurons expressing GFP (Figure 3A–G, I; Table S3). However, in four out of five embryos, we observed that LMCm neurons with elevated Dab1 expression were observed in a more ventro-lateral position (Figure 3E–I; (ML: 70%; DV: 49%)) compared to LMCm neurons expressing GFP (Figure 3A–D, I; (ML: 67%; DV: 59%), *p* = 0.0165, Hotelling's T^2^ test; Table S3), demonstrating that increasing Dab1 expression levels in LMC neurons is sufficient to shift their position laterally.

### Dissociation of Axon Trajectory from Soma Position in *Reln* and *Dab1* Mutants

**Figure 4:**
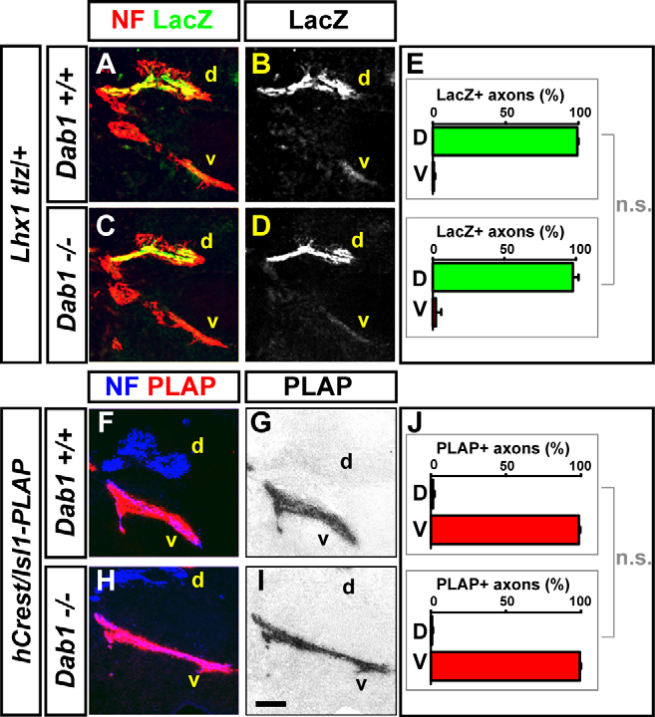
Loss of Reelin signalling disrupts LMC myotopy. (A–D) Axonal neurofilament (NF) and LacZ protein in a representative forelimb section of e11.5 control *Lhx1^tlz/+^* littermate (A–B) and *Dab1* ^−/−^; *Lhx1^tlz/+^* embryos (C–D). (E) Quantification of LacZ^+^ axons within dorsal (d) and ventral (v) limb nerves expressed as a percentage of total LacZ^+^ signal within both limb nerves. Student's *t* test *p* value: *Dab1^+/+^*; *Lhx1^tlz/+^* versus *Dab1* ^−/−^; *Lhx1^tlz/+^*, *p*≥0.5. *n* = 3 embryos per genotype; *N*>8 sections quantified per embryo. (F–I) Neurofilament (NF) immunodetection and alkaline phosphatase (PLAP) enzymatic detection in axons of a representative forelimb section of e11.5 control *hCrest/Isl1-PLAP* littermate (F–G) and *Dab1* ^−/−^; *hCrest/Isl1-PLAP* embryos (H–I). PLAP enzymatic reaction signal images (G, I) were colour-inverted and overlaid with same section NF signal images in (F, H). (J) Quantification of PLAP^+^ axons within dorsal (d) and ventral (v) limb nerves expressed as percentage of total PLAP^+^ signal within both limb nerves. Student's *t* test *p* value: *Dab1^+/+^*; *hCrest/Isl1-PLAP* versus *Dab1* ^−/−^; *hCrest/Isl1-PLAP*, *p* = 0.335. *n* = 3 embryos per genotype; *N*>6 sections quantified per embryo. Scale bar: 90 µm.

The myotopic relationship between LMC soma position and axon trajectory within the limb raises the possibility that changes in LMC soma position in *Dab1* or *Reln* mutants could result in the selection of inappropriate limb trajectory by LMC axons. To examine the LMCl axon limb trajectory in *Dab1* mutants, we used the *Lhx1^tlz^* marker line [42] and quantified the proportion of LacZ^+^ LMCl axons projecting into e11.5 forelimb dorsal and ventral limb nerves in *Dab1* ^−/−^; *Lhx1^tlz^* ^/+^, and *Lhx1^tlz/+^* littermate embryos [24]. In *Lhx1^tlz/+^* embryos we observed ∼99% of LacZ^+^ axons within the dorsal limb nerves and ∼1% of LacZ^+^ axons within the ventral limb nerves (Figure 4A, B, E). The proportions of LacZ^+^ in dorsal and ventral limb nerves of littermate *Dab1* ^−/−^; *Lhx1^tlz^* ^/+^ embryos were not significantly different (Figure 4C–E; 98% and 2%, respectively, *p*>0.5, Student's *t* test). Additionally, in whole mount e12.5 *Dab1* ^−/−^; *Lhx1^tlz/+^* embryos, we did not detect any aberrantly projecting LMCl axons at either limb level (unpublished data).

To trace LMCm axons we used the *hcrest/Isl1-PLAP* reporter line in which the *Isl1* enhancer-promoter drives the expression of placental alkaline phosphatase (PLAP) in LMCm neurons at forelimb levels [43]. PLAP enzymatic reaction was used to detect LMCm axons in *Dab1* ^−/−^; *hcrest/Isl1-PLAP^+^* and control *hcrest/Isl1-PLAP^+^* e11.5 forelimbs, followed by axonal signal quantification. In *hcrest/Isl1-PLAP^+^* embryos, ∼99% of PLAP^+^ axons were found in the ventral limb nerve, while ∼1% of PLAP^+^ axons were found in the dorsal limb nerve (Figure 4F, G, J), proportions not significantly different from *Dab1* ^−/−^; *hcrest/Isl1-PLAP^+^* embryos (Figure 4H–J; 99% and 1%, respectively; *p* = 0.335, Student's *t* test). LMCm limb trajectory in *Reln* mutants was also apparently normal (unpublished data), indicating that neither Dab1 nor Reelin are required for the selection of limb trajectory by LMC axons and demonstrating that the LMC soma position can be dissociated from axon trajectory selection.

### Foxp1 Controls Dab1 Expression in LMC Neurons

**Figure 5:**
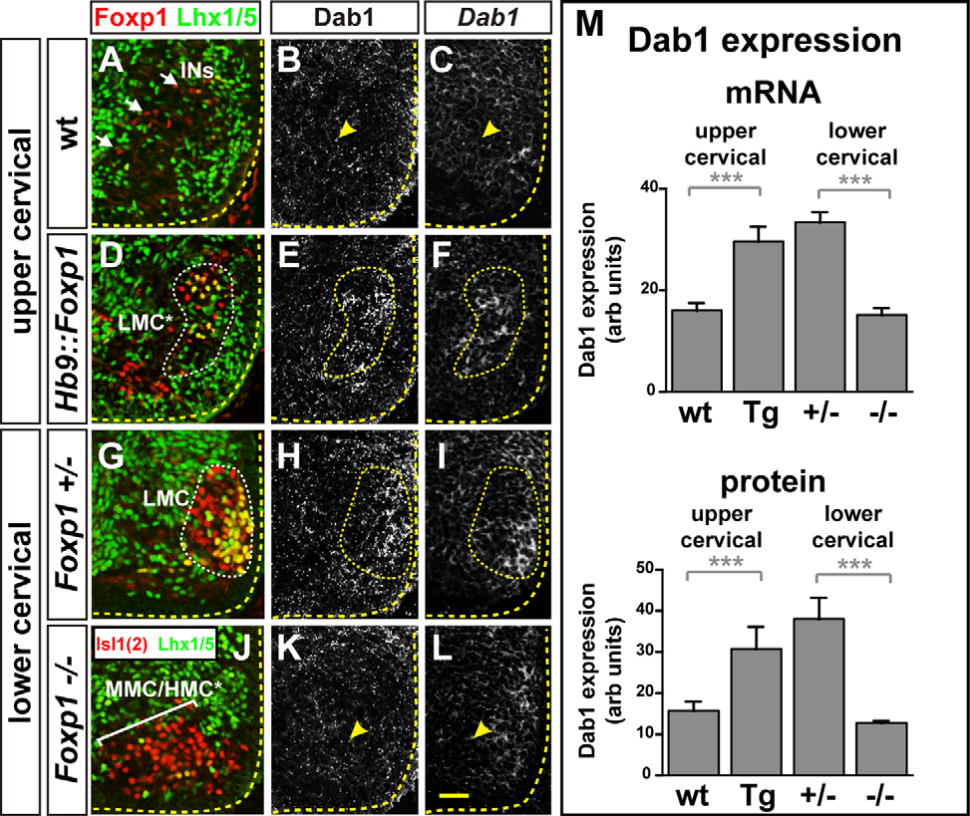
Foxp1 regulates Dab1 expression. (A–L) *Dab1* mRNA and protein expression in the cervical spinal cord of e12.5 *Hb9::Foxp1* transgenic (D–F), *Foxp1* mutant (J–L), and control embryos (A–C, G–I). (M) *Dab1* mRNA and protein mean pixel intensity measurement within Foxp1^+^ Lhx3^−^ LMC regions (fine stippled outline; D–F, G–I) or the corresponding Foxp1^−^ Lhx3^−^ region (arrowheads; B–C, K–L). arb: arbitrary units on a 0 (no signal) to 255 (maximum intensity) scale. Student's *t* test *p* values for mRNA quntifications: *Hb9::Foxp1* versus wt, *p* = 0.002, *n* = 3 embryos per genotype analysed; *Foxp1* ^−/−^ versus *Foxp1^+/^* ^−^, *p*<0.001, *n* = 3 embryos per genotype analysed; Student's *t* test *p* values for protein quantification: *Hb9::Foxp1* versus wt, *p*<0.001, *n* = 4 embryos per genotype analysed; *Foxp1* ^−/−^ versus *Foxp1^+/^* ^−^, *p*<0.001, *n* = 3 embryos per genotype analysed. All sections of the same genotypes are consecutive. Arrows: Foxp1^+^ interneurons (INs); LMC*: ectopic LMC; bracket: expanded MMC/HMC*. Thicker stippled lines outline the spinal gray matter. Scale bar: 50 µm.

Since our results indicated that the Dab1 protein level determines the position of LMC neuron somata but not their axon trajectory, we next evaluated whether the deployment of effector pathways governing these processes might be coordinated by a common set of transcriptional inputs. To determine whether Foxp1, a transcription factor specifying LMC cell fate, participates in the control of Dab1 expression in LMC neurons, we analyzed the embryonic spinal cords in which *Foxp1* is expressed in all motor neurons (*Hb9::Foxp1* transgenic) as well as in those lacking *Foxp1* function [21],[23]. We first focused our analysis on upper cervical levels, where Foxp1 and Dab1 expression levels are normally low or undetectable (Figure 5A–C; Figure S7; unpublished data). In e12.5 *Hb9::Foxp1^+^* spinal cords, compared to control embryos, we observed a significant increase in *Dab1* mRNA levels (30 arbitrary (arb.) units versus 16 in controls; *p* = 0.002, Student's *t* test; Figure 5A, C, D, F, M) as well as protein expression levels associated with ectopic Foxp1^+^ neurons, without any obvious changes in Reelin expression (Figure 5A, B, D, E, M; Figure S7; 30 arb. units versus 16 in controls; *p*<0.001, Student's *t* test). To determine whether Foxp1 is required for Dab1 expression, we examined the lower cervical spinal cord of *Foxp1* mutant mice at e12.5. When compared to controls, *Foxp1* mutant spinal cords exhibited a significant decrease in *Dab1* mRNA levels (15 arb. units versus 33 in control littermates; *p*<0.001, Student's *t* test; Figure 5G, I, J, L, M) as well as Dab1 protein levels (Figure 5G, H, J, K, M; Figure S7; 12 arb. units versus 37 in control littermates; *p*<0.001, Student's *t* test), demonstrating that Foxp1 is both sufficient and required for Dab1 expression in migrating LMC neurons.

### Control of Differential Expression of Dab1 in LMC Neurons by Isl1 and Lhx1

Although Foxp1 controls Dab1 expression, because of its uniform expression throughout the LMC, it appeared to us an unlikely determinant of the differential level of Dab1 expression in LMCl and LMCm neurons. LIM homeodomain proteins Isl1 and Lhx1 are determinants of, respectively, LMCm and LMCl neuronal fate, can influence their migration, and can control their axon trajectory by modulating Eph receptor expression (Figure S8 and Text S1; [22],[24],[25],[42]). We thus hypothesized that while Foxp1 activates Dab1 expression in all LMC neurons, Isl1 and Lhx1 have opposing effects on Dab1: (1) Isl1 lowers Dab1 expression in LMCm neurons while (2) Lhx1 elevates Dab1 expression in LMCl neurons. We tested the first of these hypotheses by electroporating Isl1 and LacZ expression plasmids, or a control LacZ expression plasmid alone into HH St 17/19 chick lumbar spinal cords and measuring changes in *Dab1* mRNA levels relative to the unelectroporated control side at HH St 29 [22]. Expression of LacZ did not affect *Isl1* or *Dab1* mRNA expression while overexpression of Isl1 significantly reduced *Dab1* mRNA expression levels in LMC neurons (Figure S9; e/u values: 1.4 for LacZ versus 0.7 for Isl1, *p*<0.001, Student's *t* test) indicating that Isl1 can suppress *Dab1* mRNA expression. To test whether Isl1 is required to control Dab1 expression, we examined the effects of siRNAs directed against *Isl1* in LMC neurons but observed no significant difference in *Dab1* expression when compared to controls (Figure S9 and Text S1). Together, these data suggest that Isl1 is sufficient but might be dispensable for the modulation of *Dab1* expression in LMC neurons.

**Figure 6:**
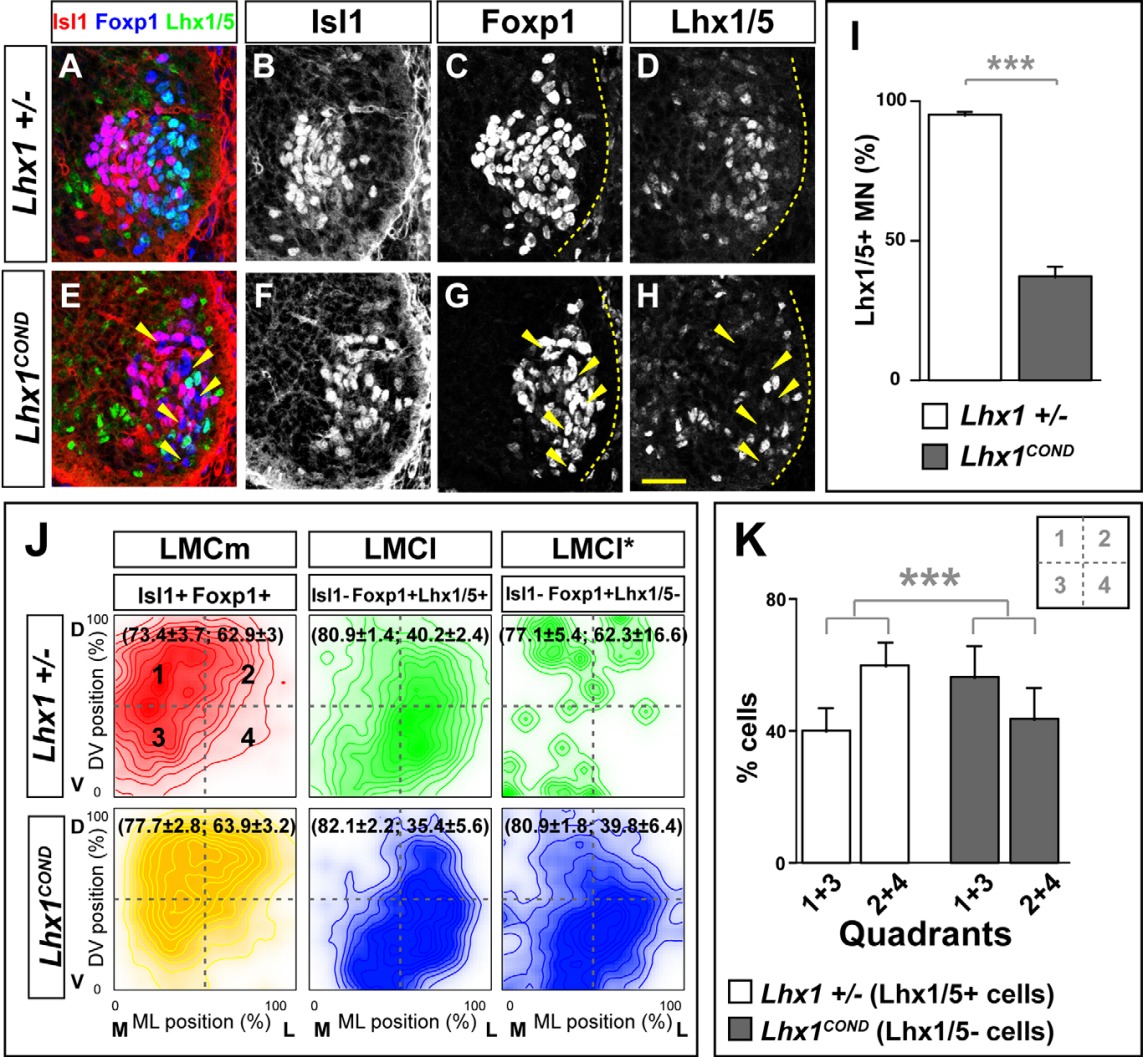
Impaired LMC migration in *Lhx1* mutants. (A–H) Foxp1, Isl1, and Lhx1/5 expression identifies LMCm (Foxp1^+^ Isl1^+^), LMCl (Foxp1^+^ Isl1^−^ Lhx1/5^+^) and LMCl* (Foxp1^+^ Isl1^−^ Lhx1/5^−^) neurons in the lumbar spinal cord of e12.5 *Lhx1* mutants (E–H), and heterozygous littermates (A–D). In *Lhx1* mutants, LMCl* cells are found more medially and intermingled with LMCm neurons (arrowheads). (I) Proportion of LMCl motor neuron in *Lhx1* mutants and control embryos expressed as percentage of Isl1^−^ Foxp1^+^ motor neurons (37.3%±9% versus 95.2%±2%; *p*<0.001, Student's *t* test). (J) Density plots of mediolateral (ML) and dorsoventral (DV) position of LMCm and LMCl neurons as percentage of LMC width and height. Mean position (ML, DV) is indicated in brackets. Hotelling's T^2^ test *p* values: LMCm(*Lhx1^+/^* ^−^) versus LMCm(*Lhx1^COND^*), *p* = 0.1613; LMCl*(*Lhx1^COND^*) versus LMCl(*Lhx1^COND^*), *p* = 0.2825; LMCl*(*Lhx1^COND^*) versus LMCl(*Lhx1^+/^* ^−^), *p* = 0.9886. (K) Proportion of LMCl and LMCl* neurons in quadrants 1–3 and 2–4 of *Lhx1^+/^* ^−^ and *Lhx1^COND^* embryos expressed as percentage of all neurons analysed. Student's *t* test *p* value: LMCl(*Lhx1^+/^* ^−^) versus LMCl*(*Lhx1^COND^*), *p* = 0.008. Dashed lines in (J) divide the plots into four equal quadrants. Stippled lines outline the spinal gray matter. *n* = 7 (*Lhx1^COND^*) and 5 (*Lhx1^+/^* ^−^) embryos; *N*>1,400 neurons per genotype. Scale bar: 50 µm.

We next tested whether Lhx1 is required to specify the position of LMCl neurons by examining embryos with a conditional loss of Lhx1 function in LMC neurons, obtained by crossing *Lhx1^flox^* homozygotes with *Isl1^Cre/+^*; *Lhx1^tlz/+^* mice, in which *Isl1^Cre^* drives Cre recombinase expression in all LMC neurons. We focused our analysis on e12.5 lumbosacral levels in two groups of embryos obtained from these crosses: *Lhx1^tlz/flox^*; *Isl1^Cre/+^*, designated as *Lhx1^COND^*, and control *Lhx1^tlz/+^*, designated as *Lhx1^+/^* ^−^. *Lhx1* loss of function did not affect the total number of LMC or LMCm neurons but resulted in ∼60% of LMCl neurons (Foxp1^+^Isl1^−^) losing their Lhx1 expression (Isl1^−^Lhx1/5^+^Foxp1^+^: 37.3% versus 95.2% in controls; *p*<0.001, Student's *t* test, Figure 6I, unpublished data). We determined the soma position of three LMC neuronal populations: LMCm, LMCl, and LMCl neurons lacking Lhx1 expression, which were defined as Isl1^−^Foxp1^+^Lhx1/5^−^ (LMCl*). As in control embryos, in which the majority of LMCl neurons settled in the most lateral part of the LMC, in *Lhx1^COND^* embryos, a significant proportion of LMCl* neurons settled laterally and the mean position of LMCm, LMCl, or LMCl* neurons was not changed when compared to controls (Figure 6A–J; Table S4). However, in *Lhx1^COND^* embryos, many LMCl* neurons were found in medial locations, intermingled with LMCm neurons (Figure 6A–H), and these neuronal displacements were more evident when we superimposed the positions of LMCl*, LMCl, and LMCm neurons in images of adjacent control and *Lhx1^COND^* spinal cords sections (Figure S10). To further characterise the medially displaced population of LMCl* neurons, we counted the number of LMC neurons in four equal quadrants of the LMC (Figure 6J, K, unpublished data). In both *Lhx1* mutant and control embryos the majority of LMCm neurons were in the medial half of the LMC (unpublished data). In control embryos, 60% of LMCl neurons were in the lateral half of the LMC, compared to 42% of LMCl* neurons in *Lhx1* mutants, representing a significant change (*p* = 0.003, Student's *t* test, Figure 6K), indicating that Lhx1 is required for LMCl position specification.

**Figure 7:**
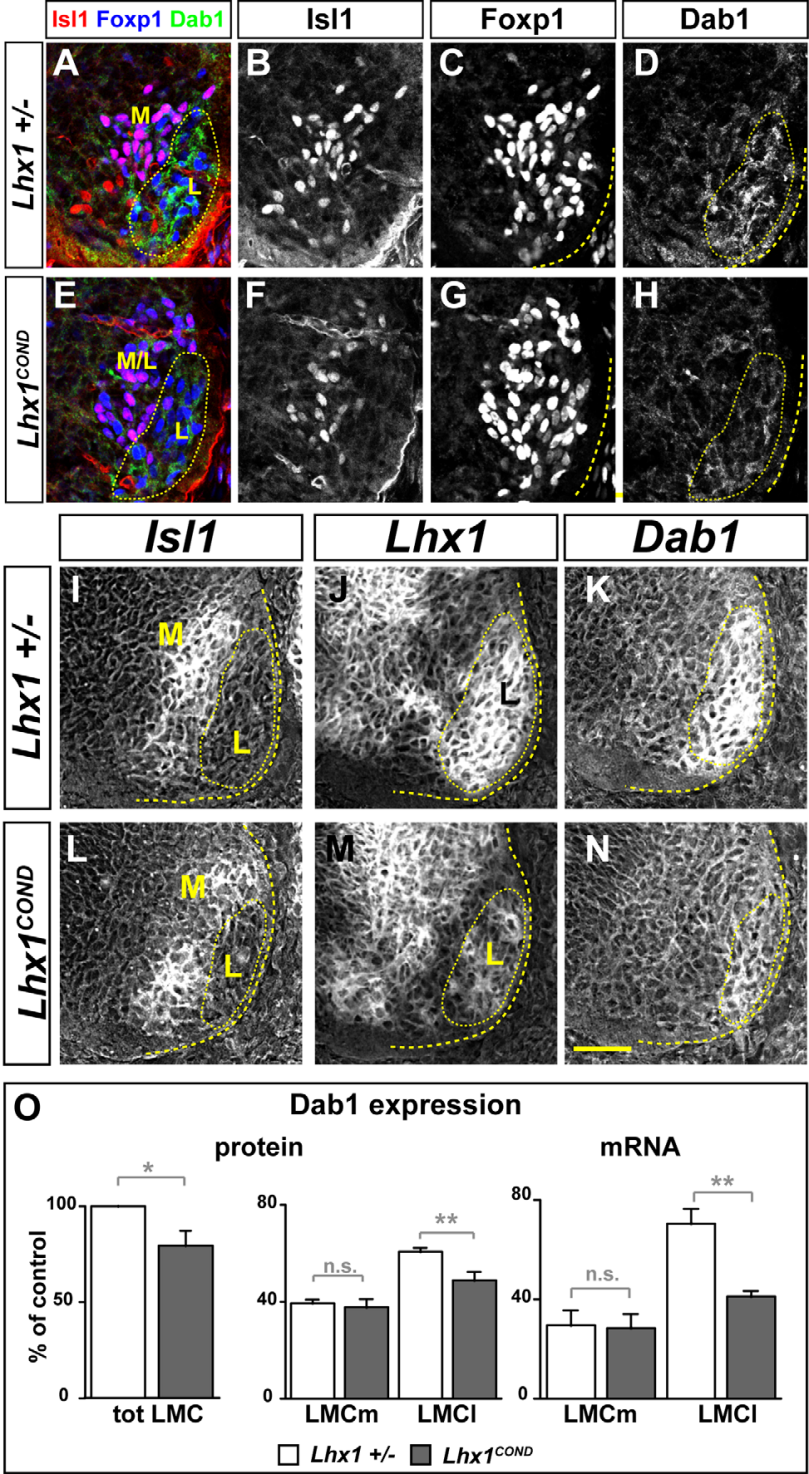
Lhx1 controls the differential expression of Dab1 in LMC neurons. (A–N) e12.5 lumbar spinal cord Dab1 protein (A–H) and mRNA (I–N) expression in LMCl (Isl1^−^ Foxp1^+^) is decreased in *Lhx1^COND^* mutants (E–H, L–N) compared to control embryos (A–D, I–K). (I–N) Detection of *Lhx1*, *Isl1*, and *Dab1* mRNA in consecutive sections of e12.5 *Lhx1^COND^* and control lumbar spinal cord. (O) Quantification of Dab1 protein and mRNA levels in LMC (Foxp1^+^), LMCm (Isl1^+^ Foxp1^+^), and LMCl (Isl1^−^ Foxp1^+^) spinal cord area normalized to Dab1 protein or mRNA levels in LMC of littermate control embryos. Dab1 protein expression in LMC of *Lhx1* mutants was 80%±16% of the expression level of control littermate embryos (*p* = 0.038; Student's *t* test; *n* = 4 embryos per genotype analysed). In heterozygous embryos LMCm Dab1 protein expression was 39%±4% and in *Lhx1* mutants was 42%±5% (*p*>0.5; Student's *t* test; *n* = 4 embryos per genotype analysed), while *Dab1* mRNA expression in *Lhx1^COND^* mutants was 29%±6% compared to 30%±6% in heterozygous embryos (*p*>0.5; Student's *t* test; *n* = 3 embryos per genotype analysed). In heterozygous embryos, LMCl Dab1 protein expression (61%±4%) and mRNA (70%±6%) was significantly different from *Lhx1^COND^* mutants (protein 47%±8%; *p* = 0.016, Student's *t* test; *n* = 4 embryos per genotype analysed; mRNA 41%±2%; *p* = 0.01, Student's *t* test; *n* = 3 embryos per genotype analysed). All values are expressed as mean ± s.d. Yellow ovals highlight LMCl neurons; yellow lines outline the spinal gray. Scale bar: 66 µm (A–H), 50 µm (I–N).

To determine whether Lhx1 directs LMCl migration by controlling Dab1 expression, we compared Dab1 protein levels in the lumbar spinal cord of e12.5 *Lhx1* mutants in which at least 50% of LMCl neurons lost their Lhx1 expression and littermate controls [22]. Our analysis revealed that in *Lhx1* mutants, Dab1 protein expression in LMC neurons was decreased by ∼20% when compared to control embryos (Figure 7A–H, O; *p* = 0.038, Student's *t* test). We also quantified *Dab1* mRNA and protein levels in the LMCm, defined as containing >90% of Isl1^+^Foxp1^+^ neurons and LMCl defined as Isl1^−^Foxp1^+^. Within the LMCm, *Dab1* mRNA and protein levels were not significantly different from controls, while in LMCl of *Lhx1* mutants, relative to controls, *Dab1* mRNA was decreased significantly by approximately 40% (*p* = 0.01, Student's *t* test) and Dab1 protein was decreased significantly by ∼14% (*p* = 0.017, Student's *t* test, Figure 7O), indicating that Lhx1 is required for the differential expression of Dab1 in LMC neurons. Together, our results reveal that Foxp1 and Lhx1 coordinate LMC myotopy through their modulation of expression of neuronal migration and axon guidance effectors.

## Discussion

Our observation that Reelin is an essential signal specifying the location of LMC neurons in the ventral spinal cord allowed us to address how neuronal migration and axon guidance are coordinated to achieve topographical organisation. Our experiments demonstrate that the transcription factors specifying the axon trajectory of LMC neurons occupy a privileged position in the molecular hierarchy controlling myotopy as they also control LMC soma migration by gating Reelin signalling. Here we discuss Reelin as a motor neuron migration signal, coordination of axon trajectory selection and soma placement, and the possible functional consequences of myotopic organisation of motor neurons.

### Reelin as a Migration Signal for Motor Neurons

Following their birth near the ventricular zone, spinal neurons first migrate radially by perikaryal translocation, then tangentially, either in dorsal or ventral direction [14]. Reelin has been proposed as a radial migration signal; however, our observations argue that the initial, apparently radial trajectory of LMC motor neurons is Reelin signalling independent as is the case of PG and hindbrain motor neurons [36],[39]. Thus, in general, the radial migration trajectory of motor neurons might not require Reelin signalling, but once it is terminated, Reelin becomes an important guidance signal, suggesting that unlike cortical neurons that rely on Reelin for their localisation in the radial plane, motor neurons at different rostrocaudal levels of the spinal cord depend on Reelin for the tangential aspect of their migration.

How does Reelin act in motor neuron migration? The initial model where Reelin is a migration stop signal has been challenged by observations that Reelin overexpression in the cortical ventricular zone can rescue, at least in part, pre-plate splitting defects associated with Reelin loss of function [34],[35]. Likewise, overexpression of Reelin in the ventricular zone of the spinal cord rescues *Reln* mutant PG neuron migration defects but does not cause an overt phenotype in a wild type background [44]. In the context of LMC neurons, the Reelin expression domain is intercalated between the emerging postmitotic neurons and their final lateral position, thus precluding a function as a migration stop signal, unless at the time of their early migration LMC motor neurons are insensitive to Reelin. Our functional Reelin fragment overexpression in the ventral spinal cord resulted in LMCl motor neurons moving beyond their normal lateral position (E.P., T.-J.K., and A.K., unpublished observations); thus, in the context of motor neurons, Reelin is unlikely to function as a migration stop signal, rather, it likely promotes migration or enables LMC neurons to respond to a cue that provides spatial information.

What is the relationship of the Reelin-mediated LMC position specification to that mediated by cadherins, Eph receptors, and the transcription factor Pea3 [27],[28],[29]? Because of their restricted expression patterns and functional analysis phenotypes, these are thought to operate at the level of motor pools, in contrast to Reelin signalling which appears to specify the position of the entire LMCl division. Cadherins have been shown to be involved in the clustering of specific motor pools via their combinatorial expression imparting different adhesion properties on specific motor pools. Similarly, although the early migration of LMC motor neurons in *EphA4* mutants appears to be normal, eventually the position of the tibialis motor pool is shifted. Because of these observations, it is likely that Cadherins, EphA4, and Pea3 act at a step following Reelin-mediated migration of LMCl neurons. Unfortunately, since ETS genes, arguably the earliest molecular markers of motor pools, begin to be expressed at the time when LMCl somata attain their lateral position [45], it is technically difficult to ascertain experimentally whether motor pool clustering precedes or coincides with LMCl lateral migration. The differences between the LMC position phenotypes in *Dab1* and *Lhx1^COND^* mutants might shed some light on this hierarchy. In *Dab1* mutants, although shifted medio-ventrally, LMCl neurons remain clustered, in contrast to *Lhx1* mutant LMCl motor neurons that can be found intermingled with LMCm neurons. These observations suggest that while the *Dab1* mutation probably only leads to the absence of sensitivity to Reelin, the loss of the transcription factor Lhx1 might have consequences beyond the loss of Dab1, resulting, for example, in a change in expression of cell surface adhesion molecules allowing LMCl and LMCm neurons to intermingle.

### Dab1 as a Neuronal Position Determinant

Our findings demonstrate that migration of LMC neurons within the ventral spinal cord requires Reelin signalling through the intracellular adaptor protein Dab1. This requirement is principally evident in LMCl neurons and corresponds to the high level of Dab1 protein and mRNA expressed in this population when compared to LMCm neurons. Other studies have also implicated Dab1 protein levels controlled by Cullin5 and Notch signalling as a determinant of neuronal migration [46],[47], raising the question of how might differential Dab1 expression specify LMC soma position in the ventral spinal cord. Upon activation of the Reelin pathway, Dab1 is phosphorylated and rapidly degraded [30],[34]. Therefore, in the presence of Reelin, the low Dab1 protein levels in LMCm neurons might be depleted faster than the higher Dab1 protein levels in LMCl neurons, resulting in the termination of Reelin signalling and thus a migration stop occurring sooner in LMCm neurons than in LMCl neurons. This mode of Dab1 function assumes that Reelin promotes migration of LMC neurons, or is a factor enabling their reception of a migration cue and is consistent with our observation that both LMCl and LMCm neurons can respond to Reelin. Thus similar to the Toll-like receptor (TLR) [48] and chemokine [49] signalling pathways regulated by the level of expression of a signalling intermediate, Reelin signal is differentially gated in two neuronal populations through opposing levels of Dab1 expression. In such a model, we would favour the idea that Dab1 concentration, in the presence of Reelin, is an instructive determinant of LMC neuron position, although the formal demonstration of this through, for example, the change of LMCm Dab1 levels to match exactly those in LMCl neurons is technically challenging. Following its phosphorylation, Dab1 is targeted for polyubiquitination and degradation by Cullin5 [47], raising the possibility that in LMC neurons, Dab1 protein stability might contribute to the differences in Dab1 protein in LMC neurons. However, since in LMC neurons Cullin5 is apparently expressed at equal levels by LMCl and LMCm neurons (E.P. and A.K., unpublished observations), and because of the selective enrichment of *Dab1* mRNA in LMCl neurons, compared to LMCm neurons, we favour the hypothesis that differential transcriptional regulation of the *Dab1* gene or its mRNA stability is an important factor contributing to Dab1 protein levels in LMC neurons.

### Gating of Reelin Signalling by Transcription Factor Restriction of Dab1 Expression

Our results demonstrate that Dab1 expression levels in LMC neurons are set by Foxp1 and Lhx1, two transcription factors that are essential for the specification of LMC soma position [21],[22],[23]. Our data suggest the following model of Dab1 expression control in LMC neurons: a basal level of Dab1 expression in LMC neurons is induced or maintained by Foxp1, while Lhx1, a transcription factor selectively expressed in LMCl neurons, could act to elevate Dab1 expression in LMCl neurons. Additionally, based on its ability to suppress Lhx1 [22] and *Dab1* mRNA expression in LMC neurons, Isl1 might function to diminish Dab1 expression in LMCm neurons. Thus, although we cannot exclude the influence of other transcription factors or distinguish whether the control of Dab1 expression by Foxp1 and Lhx1 occurs at the level of the *Dab1* promoter, through intermediary transcription factors or regulation of *Dab1* mRNA stability, we propose that the concerted action of Foxp1 and Lhx1 leads to differential Dab1 expression levels in LMC neurons.

Could transcription factor control of Dab1 expression be a general mechanism gating Reelin signalling in the CNS? In the cortex, examples of control of migration effectors by transcription factors include the coupling of neurogenesis to migration by bHLH control of doublecortin and p35, Tbx20 control of the planar cell-polarity pathway, and Nkx2.1 control of Neuropilin2 expression [6], but to our knowledge, a general link between a specific transcription factor and Dab1 expression has so far only been established for CREB/CREM [50]. Intriguingly, in the spinal cord, like LMC neurons, PG neurons migrate in response to Reelin and also require Foxp1 for their specification [21],[23],[36], yet although their initial lateral migration path is shared, they eventually occupy two distinct locations in the spinal cord, raising the question of the identity of the divergent migration cues that act on these two motor neuron populations.

### Coordination of Myotopy by Transcription Factors

**Figure 8:**
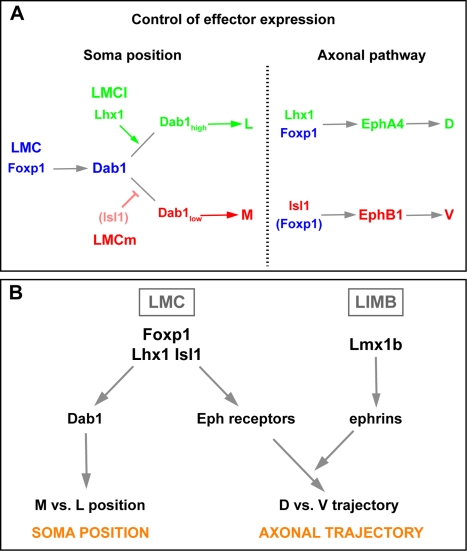
Model of transcriptional control of LMC myotopy. (A) Foxp1, Isl1, and Lhx1 control Dab1 and Eph receptor expression in LMC neurons and thus coordinate LMC neuronal migration and axon projections. (B) Molecular hierarchy of transcription factor control of LMC myotopy. See discussion for details.

The myotopic organisation of spinal motor neurons is the consequence of the selection of a specific axon trajectory in the limb mesenchyme and of a particular soma location within the spinal cord. The two processes can be uncoupled by loss of Reelin, Eph signalling, or mutation of Lmx1b, a LIM homeodomain transcription factor that controls ephrin ligand expression in the limb [24],[26],[42], raising the question of the molecular hierarchy controlling myotopy. Foxp1 and Lhx1 determine the selection of a dorsal or ventral LMC axon trajectory through restriction of Eph receptor expression [21],[22],[23], and our data suggest that they gate LMC neuron sensitivity to Reelin signals, thereby specifying the position of LMC soma in the ventral spinal cord. These observations imply that the selection of an LMC axon trajectory in the limb and soma position within the ventral horn are normally controlled coordinately by Foxp1 and LIM homeodomain transcription factors. Based on these observations, we propose a simple hierarchy for motor axon trajectory and soma position selection coordination (Figure 8). Foxp1 together with Lhx1 and Isl1 transcription factors are required for the expression of Eph receptors in LMC axons, and thus their repulsion from ephrin ligands in the limb mesenchyme, leading to their selection of a dorsal or a ventral limb trajectory. Foxp1, Lhx1, and possibly Isl1 also establish disparate Dab1 protein levels in LMC neurons, thus enabling their cell bodies to segregate into distinct mediolateral positions. A number of transcription factors regulating reception of specific axon guidance receptors has already been described [4],[5], implying that some of them may also direct neuronal migration, thus coordinating topographic organisation of neuronal circuits. Moreover, topographical organisation also extends to dendrite arborisation and synaptic activity [19],[51], and since Foxp1 regulates the position of motor neuron dendrites [21], it remains plausible that the transcription factors controlling migration and axon projections may be used to control other facets of topographic organisation.

Why should neuronal migrations and axon trajectories be controlled coordinately? LMC neurons within a specific motor pool, i.e. those innervating a particular muscle, are electrically coupled through gap junctions, possibly to consolidate their electrical activity patterns during the time of spinal motor circuit assembly [52]. Aberrant soma position could result in the inability of LMC neurons to form electrically coupled motor pools even though neuromuscular junctions with appropriate muscle targets in the limb might be maintained. Thus, a motor neuron might receive appropriate signals from its muscle target but is unable to synchronise its electrophysiological maturation, such as calcium transient waves [53], with other motor neurons in its pool because of their dispersed position. The emergence of functional motor circuitry also depends on the formation of specific sensory-motor contacts achieved by sensory axons synapsing on the dendrites of homonymous motor neurons within the ventral spinal cord [54]. Motor neurons in distinct pools have stereotypic dendritic arbor shapes which in principle could be dictated by the position of the motor neuron soma [28], although it remains to be determined whether motor neuron soma displacement, without any effects on molecular markers of cell fate, results in dendritic arborisation defects and whether such defects alter the sensory-motor connectivity. Reelin signalling has also been implicated in cortical dendrite formation, raising the possibility that *Reln* mutation might lead to LMC dendritic arbour defects independently of its effect on soma localisation. Moreover, in *Reln* mutant mice, although retrograde and electrophysiological analysis reveals relatively normal cortico-thalamic connectivity, retinal circuit connectivity is perturbed possibly due to defects in neuronal layer formation [55],[56]. Because of the involvement of Reelin in synapse function [57], it is difficult to dissociate the functional consequences of altered topography in Reelin signalling loss of function from altered synaptic function. However, examples of severe functional deficits caused by neural circuit topography disruption apparently independent of Reelin signalling [58] highlight the importance of topographic organisation of the nervous system.

## Materials and Methods

### Animals

All mice were maintained and genotyped by PCR as previously described [21],[31],[43],[59],[60],[61],[62]; *Reln* allele was *Reln^rl/J^* (Jackson Laboratory, USA). Fertilised chick eggs (Couvoir Simentin, Canada) were staged according to Hamburger and Hamilton [38].

### Expression Plasmid Generation and Chick In Ovo Electroporation

Chicken Dab1L isoform (NM_204238) [63] was cloned by RT-PCR (Invitrogen, USA) and fused in frame to GFP at the C-terminus in pN2-eGFP (Invitrogen, USA).

Chick spinal cord electroporation was performed using a Ovodyne TSS20 square pulse generator (Intracell, UK) as described [24],[64].

### Immunostaining and In Situ mRNA Detection

Immunofluorescence stainings were carried out on 12 µm cryosections as described [22],[24]. For antisera used and dilutions, see Table S1.

In situ mRNA detection was performed as previously described [65],[66]. Probe sequence details are available upon request.

### Image Quantification

Images were acquired using a Zeiss LSM confocal microscope or a Leica DM6000 microscope with Improvision Volocity software. Quantification of protein and mRNA expression, GFP- and β-gal-labelled axon projections was as described [24],[65]. To quantify axon projections in *hCrest/Isl1-PLAP* embryos, 12 µm cryosections were immunostained (see Table S1), post-fixed, washed, and incubated at 65°C. Phosphatase activity was revealed simultaneously in sections containing mutant and control tissue. The signal was quantified in sections sampled at 30–50 µm rostrocaudal intervals at the cervical level with at least six sections analysed per embryo.

### Motor Neuron Position Quantification

All quantifications were done between lumbosacral (LS)4 and LS6 levels as assessed by vertebra counts and absence of pSmad1^+^ PG neurons [23]. Neurons were imaged in 12 µm cryosections sampled at 100 µm intervals using a Zeiss LSM confocal or Leica DM6000 fluorescent light microscope; ML and DV values were calculated using ImageJ software measurements of distance (D) and angle (α) of motor neuron soma from the ventral edge of the ventricular zone (see Text S1 for details) and then plotted using Matlab software running the “dscatter” function, which creates a scatter plot with contour lines linking data points with similar frequency and colour intensities that increase with data point frequency.

In all cases, to compare the vectors of means between experimental and control groups, we used a two-sample Hotelling's T^2^, which is a two-dimensional generalization of the Student's *t* test, combined with a randomization test under the assumption of unequal variances, which does not rely on the stringent assumptions of the parametric Hotelling's T^2^, to circumvent the difficulty of having moderately sized samples. The analysis was implemented using the NCSS software package (Hitze J. (2007); Kaysville, Utah, www.ncss.com).

## Supporting Information

Figure S1 **Expression of Dab1 and Reelin in e10.5 mouse and** ***Dab1*** **,** ***Reelin*** **,** ***VLDLR*** **, and** ***ApoER2*** **in the chick spinal cord.** (A–L) Detection of Isl1, Lhx1/5, Foxp1, and Dab1 in cervical (A–D) and lumbar spinal cord (E–H) and Reelin in cervical spinal cord (I–L). Dab1 is expressed in Foxp1^+^ LMC neurons, while Reelin expression is restricted to a small domain dorsal to the LMC. (M–P, R–U) Detection of mRNA in consecutive lumbar spinal cord sections of HH St 24 and HH St 30 chick embryos. *Isl1* (M, R) and *Lhx1* (N, S) expression highlights LMCm and LMCl neurons, respectively. *Reelin* mRNA is expressed medio-dorsally to the LMC at HH St 24 (P), but at HH St 30 this expression domain is expanded medio-ventrally (U). Detection of *Dab1* mRNA expression is stronger in LMCl neurons compared to LMCm neurons at both HH St 24 (O) and HH St 30 (T). (W–Z) Detection of *Isl1* (W), *Lhx1* (X), *ApoER2* (Y), and *VLDLR* (Z) mRNA in HH St 25/26 chick embryos. *ApoER2* mRNA is present in the ventricular zone (VZ) but not in LMC neurons (Y). *VLDLR* expression is uniform throughout the ventral spinal cord (Z). (Q, V) Schematised LMCm and LMCl position within ventral spinal cord. VZ: ventricular zone; yellow stippled lines outline the spinal gray. Scale bar: 62 µm (A–L), 52 µm (M–U), and 59 µm (W–Z).
(4.26 MB TIF)

Figure S2 **Expression of RALDH2, RC2, and Cadherins in** ***Dab1*** **mutants.** (A–L) Protein expression in lumbar spinal cord of e12.5 embryos. Foxp1 expression highlights LMC neurons; LMCm (M) neurons are Foxp1^+^ Isl1^+^, LMCl (L) neurons are Foxp1^+^ Isl1^−^. In *Dab1* mutants, RALDH2 (A–F) and RC2 (G–L) distribution is unchanged when compared to control embryos. (M–X) Detection of cadherin mRNAs in consecutive sections of e12.5 lumbar spinal cord of *Dab1* mutants (S–X) and control littermate (M–R). *Isl1* (M, S) and *Lhx1* (N, T) expression highlights LMCm (M) and LMCl (L) motor neurons, respectively. Scale bar: 50 µm (A–L) and 84 µm (M–X).
(5.77 MB TIF)

Figure S3 **Quantification of motor neurons in** ***Dab1*** **and** ***Reln*** **mutant embryos.** (A, C) Quantification of LMC motor neuron numbers in e12.5 *Dab1*, *Reln*, and wild type (wt) littermate embryos expressed as the average number of total Foxp1^+^ LMC neurons per 12 µm section (# Foxp1+/section). LMC neuron numbers are not significantly different in mutants when compared to controls (*p*>0.17, Student's *t* test). (B, D) Quantification of LMCm (Isl1^+^ Foxp1^+^) and LMCl (Isl1^−^ Foxp1^+^) motor neuron numbers in lumbar spinal cord of e12.5 *Dab1* (B) and *Reln* (D) mouse embryos expressed as the percentage of all LMC motor neurons in 12 µm sections (Foxp1+ MN (%)). LMCm and LMCl neuron numbers are not significantly different in mutants when compared to controls (*p*>0.25 Student's unpaired *t* test).
(0.32 MB TIF)

Figure S4 **Characterisation of Dab1 and Reelin mutants.** (A–F) Loss of Dab1 immunoreactivity (arrowheads) in the lumbar spinal cord of an e12.5 *Dab1* mutant embryo (D–F) compared to a control littermate (A–C). LMCl (L) neurons are Foxp1^+^ Isl1^−^ and LMCm (M) neurons are Foxp1^+^ Isl1^+^. (G–L) Loss of Reln protein immunoreactivity (arrows) in the lumbar spinal cord of a e12.5 *Reln* mutant embryo (J–L) compared to a control littermate (G, I). Dab1 protein levels are increased in the *Reln* mutant (J, K) relative to the control littermate (G, H) (arrowheads). Isl1 expression highlights LMCm neurons. Asterisks indicate blood vessels. Stippled lines outline the spinal gray or LMCl (L) neurons. Scale bar: 50 µm in all panels.
(4.36 MB TIF)

Figure S5 **Cell autonomous requirement for Dab1 signaling in LMCl migration.** (A) Quantification of LMC motor neurons electroporated with LacZ or Dab1^5YF^ and LacZ expression plasmids in chick HH St 29 embryos, expressed as the average number of the total LacZ^+^ LMC neurons per 12 µm section (# LacZ+ MN/section). The difference in numbers between control and experimental embryos is not significant (n.s.; *p* = 0.440, Student's unpaired *t* test; *n* = 7 embryos; *N*>100 neurons per embryo per experimental condition analysed). (B) Quantification of LMCm (Isl1^+^ Foxp1^+^) and LMCl (Isl1^−^ Foxp1^+^) motor neurons in lumbar spinal cord of chick HH St29 embryos electroporated with LacZ or Dab1^5YF^ and LacZ expression plasmids, expressed as the percentage of total electroporated motor neurons [LacZ+ MN (%)]. The difference in numbers between control and experimental embryos is not significant (n.s.; *p* = 0.393, Student's unpaired *t* test; *n* = 7 embryos; *N*>100 neurons per embryo per experimental condition analysed). (C–J) Detection of LacZ, Foxp1, and Isl1 protein in LMCm (Foxp1^+^ Isl1^+^) and LMCl (Foxp1^+^ Isl1^−^) neurons in lumbar spinal cord of chick HH St 29 embryos electroporated with LacZ (C–F) or Dab1^5YF^ and LacZ expression plasmids (G–J). LMCl neurons expressing Dab1^5YF^ are frequently found in a zone ventromedial to LMCm neurons (arrowheads in J). Examples of electroporated LMCl neurons are indicated by arrowheads (G–J) and are shown at higher magnification (insets of G–J). (K) Density plots of mediolateral (ML) and dorsoventral (DV) position of electroporated LMCm and LMCl neurons expressed as percentage of LMC width and height. The position of LacZ-expressing LMCm neurons was not significantly different from Dab1^5YF^-expressing LMCm embryos ((ML: 58.6%±2.9%; DV: 50.8%±5.5%) versus (ML: 58.1%±3.3%; DV: 50.2%±7.8%); *p* = 0.9235, Hotelling's T^2^ test). Dab1^5YF^-expressing LMCl neurons were shifted significantly in a medial direction with respect to LacZ-expressing LMCl neurons ((ML: 72.1%±3.6%; DV: 42.2%±8%) versus (ML: 78.3%±3.3%; DV: 45%±3.4%); *p* = 0.0233, Hotelling's T^2^ test). Number of embryos analysed per experimental condition: 7; number of electroporated neurons analysed per experimental condition: >100 per embryo. Dashed lines divide the LMC into four equal quadrants (I). All values are expressed as mean ± s.d. Yellow stippled lines outline the spinal gray matter. Scale bar: 23 µm in all panels.
(1.80 MB TIF)

Figure S6 **Quantification of Dab1 and LMC neuronal identity in embryos over-expressing Dab1.** (A) Quantification of LMCm (Isl1^+^ Foxp1^+^) and LMCl (Foxp1^+^ Isl1^−^) neuron numbers in the lumbar spinal cord of chick HH St 29 embryos expressing GFP or Dab1::GFP, expressed as the percentage of total motor neurons (Foxp1+ MNs (%)). The difference between experimental and control embryos is not significant (*p* = 0.4324, Student's unpaired *t* test; *n* = 4 (GFP) and 5 (Dab1::GFP) embryos; *N*>60 neurons per embryo per experimental condition analysed). (B) Proportions of electroporated LMCm (Isl1^+^ Foxp1^+^ GFP^+^) and LMCl (Isl1^−^ Foxp1^+^ GFP^+^) motor neurons in lumbar spinal cord of chick HH St29 embryos expressing GFP or Dab1::GFP (GFP+ MN (%)). The difference between experimental and control embryos is not significant (n.s.; *p* = 0.0510, Student's unpaired *t* test; *n* = 4 (GFP) and 5 (Dab1::GFP) embryos; *N*>90 neurons per embryo per experimental condition analysed). (C) In embryos electroporated with Dab1::GFP, *Dab1* mRNA is upregulated on the electroporated side with respect to the unelectroporated side of the spinal cord. Quantification of *Dab1* mRNA expression in GFP (white bar) or Dab1::GFP electroporated embryos (grey bar), normalized to the expression in the unelectroporated side of the spinal cord (electroporated/unelectroporated ratio: e/u). E/u ratio for *Dab1* mRNA expression in GFP electroporated embryo is 1±0.04, while in Dab1::GFP electroporated embryos it is 1.3±0.03 (*p*<0.001; Student's *t* test; *n* = 3 embryos per experimental condition analysed).
(0.59 MB TIF)

Figure S7 **Normal Hoxa5, Hoxc6, Reelin, and Chx10 expression in** ***Hb9::Foxp1*** **transgenics and** ***Foxp1*** **mutants.** (A, D, G, J) Hoxa5 and Hoxc6 protein detection in cervical spinal cord of *Hb9::Foxp1* (D), *Foxp1* mutant (J), and control littermate (A, G) e12.5 embryos. (B, E, H, K) Foxp1 and Hb9 detection in cervical spinal cord of *Hb9::Foxp1* (E), *Foxp1* mutant (K), and control littermate (B, H) e12.5 embryos. (C, F, I, L) Reelin and Chx10 detection in forelimbs of *Hb9::Foxp1* (F), *Foxp1* mutant (L), and control littermates (C, I) e12.5 embryos. LMC* indicates ectopic LMC in upper cervical sections of *Hb9::Foxp1* embryos.
(1.96 MB TIF)

Figure S8 **Isl1 is required for the specification of LMCm position.** (A–D, F–I) Detection of LacZ, Foxp1, and Lhx1/5 protein in LMCm (Foxp1^+^ Lhx1/5^−^) and LMCl (Foxp1^+^ Lhx1/5^+^) neurons in lumbar spinal cord of chick HH St 29 embryos electroporated with LacZ (A–E) or [Isl1]siRNA and LacZ expression plasmids (F–J). Arrowheads point to electroporated LMCm neurons. Examples indicated by arrowheads are shown at higher magnification in insets of (A–D, F–I). (E, J) Superimposed ventral spinal cord location of electroporated LMCm (red) neurons in consecutive sections of representative embryos highlights the laterally shifted position of [Isl1]siRNA and LacZ expression plasmids electroporated LMCm neurons. The blue dashed line indicates the 50% ML value. (K) Density plots of mediolateral (ML) and dorsoventral (DV) positions of electroporated LMCm and LMCl neurons expressed as percentage of LMC width and height. The mean position of [Isl1]siRNA-electroporated LMCm neurons (ML: 67.9%±1.5%; DV: 53%±6%) was significantly shifted laterally with respect to the mean position of LacZ-expressing LMCm neurons ((ML: 63.5%±1.8%; DV: 56.9%±3.8%); *p* = 0.0473, Hotelling's T^2^ test). Number of embryos analysed per experimental condition: 4 (LacZ) and 5 (LacZ+ [Isl1]siRNA); number of neurons included in the analysis: >60 per embryo per experimental condition. Dashed lines divide LMC in four equal quadrants. All values are expressed as mean ± s.d. Yellow stippled lines outline the spinal gray matter. Scale bar: 23 µm in all panels.
(1.37 MB TIF)

Figure S9 **Isl1 is sufficient to regulate** ***Dab1*** **mRNA expression.** (A–L, N–Y) *LacZ*, *Isl1*, *Lhx1*, and *Dab1* mRNA detection in chick HH St 28–30 embryos electroporated with LacZ (A–F), Isl1 and LacZ expression plasmids (G–L), GFP (N–S), or [Isl1]siRNA and GFP expression plasmid (T–Y). In embryos electroporated with Isl1, Dab1 is downregulated on the electroporated side (L) with respect to the unelectroporated side of the spinal cord (K). No significant effect on Dab1 expression was observed in LacZ electroporated embryos (E, F). (M) Quantification of *Isl1* and *Dab1* mRNA expression in LacZ (white bars) or Isl1 electroporated embryos (grey bars), with values normalized to expression in the unelectroporated side of the spinal cord (electroporated/unelectroporated ratio: e/u). E/u ratio for *Isl1* mRNA expression in LacZ electroporated embryo is 1.2±0.1, while in Isl1 electroporated embryos it is 3±0.5 (*p* = 0.002; Student's *t* test). *Dab1* mRNA expression e/u ratio in Isl1 electroporated embryos (0.7±0.1) is significantly different from that in LacZ electroporated embryos (1.4±0.1; *p*<0.001, Student's *t* test). (Z) Quantification of numbers of Isl1 or Foxp1 expressing neurons in GFP- (white bars) or [Isl1]siRNA-electroporated embryos (grey bars), expressed as the ratio between electroporated/unelectroporated spinal cord sides (e/u). E/u value for Isl1^+^ Foxp1^+^ neurons in GFP electroporated embryos is 1±0.03, and in [Isl1]siRNA electroporated embryos it is 0.8±0.05 (*p*<0.001; Student's *t* test). Foxp1^+^ neurons in [Isl1]siRNA electroporated embryos (0.95±0.0.3) is not significantly different from GFP electroporated embryos (1±0.0.2; *p*<0.075, Student's *t* test). All values are expressed as mean ± s.e.m. Fine stippled lines highlight Dab1^high^ area, heavy stippled lines outline the spinal gray. Number of embryos analysed per experimental condition: 3. Scale bar: 59 µm (A–L) and 46 µm (N–Y).
(1.82 MB TIF)

Figure S10 **Variability of LMCl neuron location in Lhx1 mutants.** Superimposed spinal cord position of LMCm (red), LMCl (green), and LMCl* (blue) neurons in consecutive sections of analyzed embryos highlights altered position of LMCl* neurons. Blue numbers indicate the fractions of LMCl* neurons expressed as percentage of Isl1^−^ Foxp1^+^ motor neurons and the dashed line represents the 50% ML value.
(1.59 MB TIF)

Table S1 **Antibodies used in this study.** (A) Primary antibodies, (B) fluorochrome-conjugated secondary antibodies. DSHB: Developmental Studies Hybridoma Bank, developed under the auspices of the NICHD and maintained by The University of Iowa, Department of Biological Sciences, Iowa City, IA 52242.
(0.09 MB DOC)

Table S2 **Position of LMC neurons in** ***Dab1*** **and** ***Reln*** **mutants.** n: number of embryos analysed; N: total numbers of neurons counted; *p* values for position of mutant versus littermate wild type neurons are from the randomized Hotelling's T^2^ test under unequal variances. ^a^ Values are ± standard deviation of the mean.
(0.04 MB DOC)

Table S3 **Position of LMC neurons in electroporated chick embryos.** n: number of embryos analysed; N: total numbers of neurons counted; *p* values for position of experimental versus control neurons are from randomized Hotelling's T^2^ test under unequal variances. ^a^ Values are ± standard deviation of the mean; ^b^ only electroporated, LacZ^+^, or GFP^+^ LMC neurons were included in the analysis; ^c^ only electroporated LacZ^+^ Lhx1/2^−^ LMC neurons were included in the analysis.
(0.06 MB DOC)

Table S4 **Position of LMC neurons in** ***Lhx1*** **mutants.** LMCm (A), LMCl and LMCl* (B) position analysis. n: number of embryos analyzed; N: total numbers of neurons counted; *p* values for position versus littermate *Lhx1 ^+/−^*, Randomization Hotellings T^2^ test under unequal variances; *p*′ values for position LMCl* versus LMCl same genotype, Randomization Hotellings T^2^ test under unequal variances; *p*″ values for position LMCl* versus LMCl littermate *Lhx1^+/^* ^−^, Randomization Hotellings T^2^ test under unequal variances. ^a^ Values are ± standard deviation of the mean.
(0.05 MB DOC)

Text S1 **Supplemental data and supplemental materials and methods.**
(0.05 MB DOC)

## Abbreviations

arb: arbitrary
HH: Hamburger and Hamilton
LMC: lateral motor column
LMCl: lateral LMC
LMCm: medial LMC
LS: lumbo-sacral
PG: preganglionic
PLAP: placental alkaline phosphatase
St: stage
wt: wild type
