# Supplementary figures and images for: Foxp1 and Lhx1 Coordinate Motor Neuron Migration with Axon Trajectory Choice by Gating Reelin Signalling
Source: PLoS Biol. 2010 Aug 10;8(8):e1000446. doi: 10.1371/journal.pbio.1000446 (PMC2919418; doi:10.1371/journal.pbio.1000446)

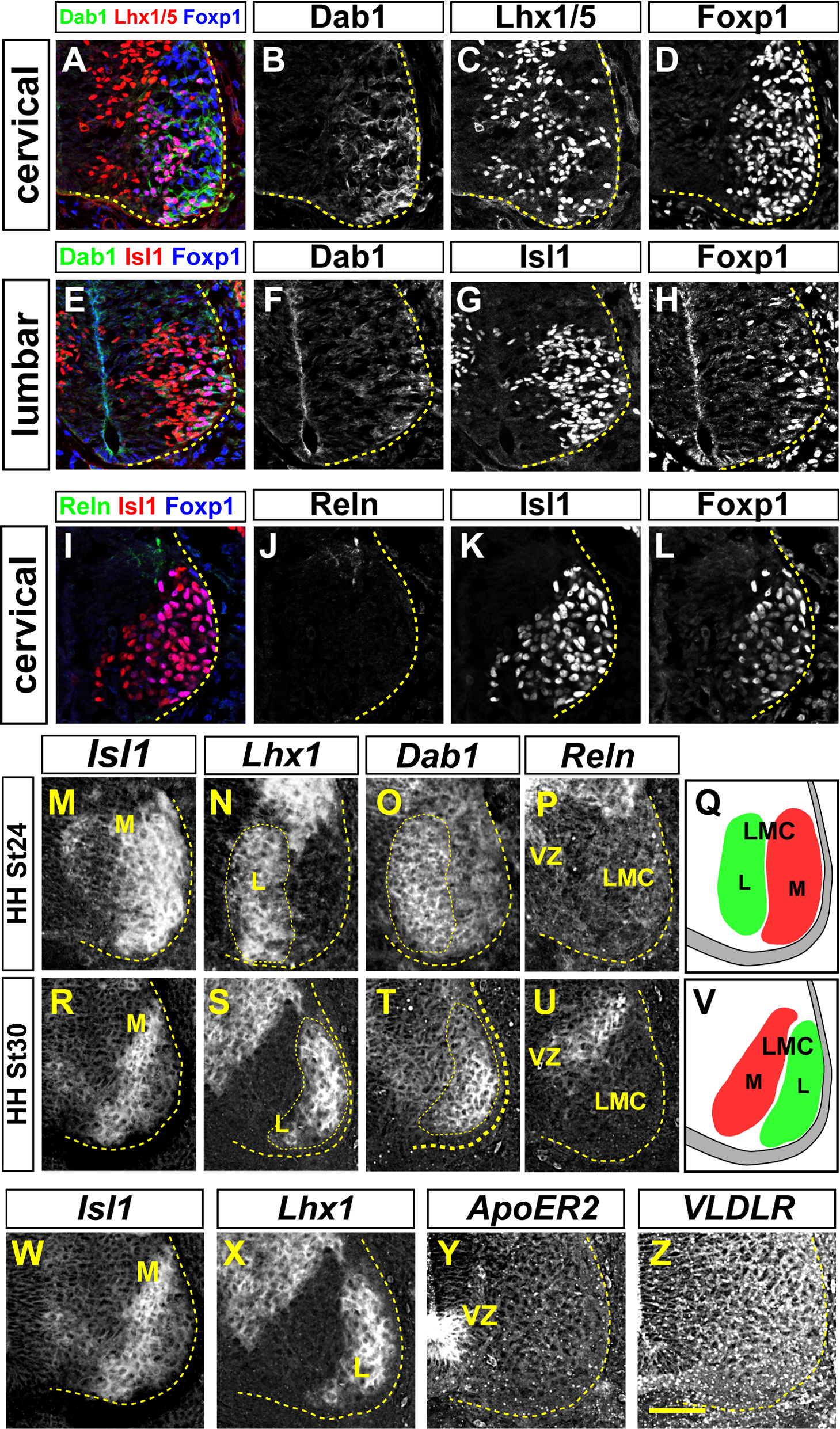

Supplement: Figure S1 — Expression of Dab1 and Reelin in e10.5 mouse and Dab1 , Reelin , VLDLR , and ApoER2 in the chick spinal cord. (A–L) Detection of Isl1, Lhx1/5, Foxp1, and Dab1 in cervical (A–D) and lumbar spinal cord (E–H) and Reelin in cervical spinal cord (I–L). Dab1 is expressed in Foxp1+ LMC neurons, while Reelin expression is restricted to a small domain dorsal to the LMC. (M–P, R–U) Detection of mRNA in consecutive lumbar spinal cord sections of HH St 24 and HH St 30 chick embryos. Isl1 (M, R) and Lhx1 (N, S) expression highlights LMCm and LMCl neurons, respectively. Reelin mRNA is expressed medio-dorsally to the LMC at HH St 24 (P), but at HH St 30 this expression domain is expanded medio-ventrally (U). Detection of Dab1 mRNA expression is stronger in LMCl neurons compared to LMCm neurons at both HH St 24 (O) and HH St 30 (T). (W–Z) Detection of Isl1 (W), Lhx1 (X), ApoER2 (Y), and VLDLR (Z) mRNA in HH St 25/26 chick embryos. ApoER2 mRNA is present in the ventricular zone (VZ) but not in LMC neurons (Y). VLDLR expression is uniform throughout the ventral spinal cord (Z). (Q, V) Schematised LMCm and LMCl position within ventral spinal cord. VZ: ventricular zone; yellow stippled lines outline the spinal gray. Scale bar: 62 µm (A–L), 52 µm (M–U), and 59 µm (W–Z). (4.26 MB TIF) [file pbio.1000446.s001.tif]

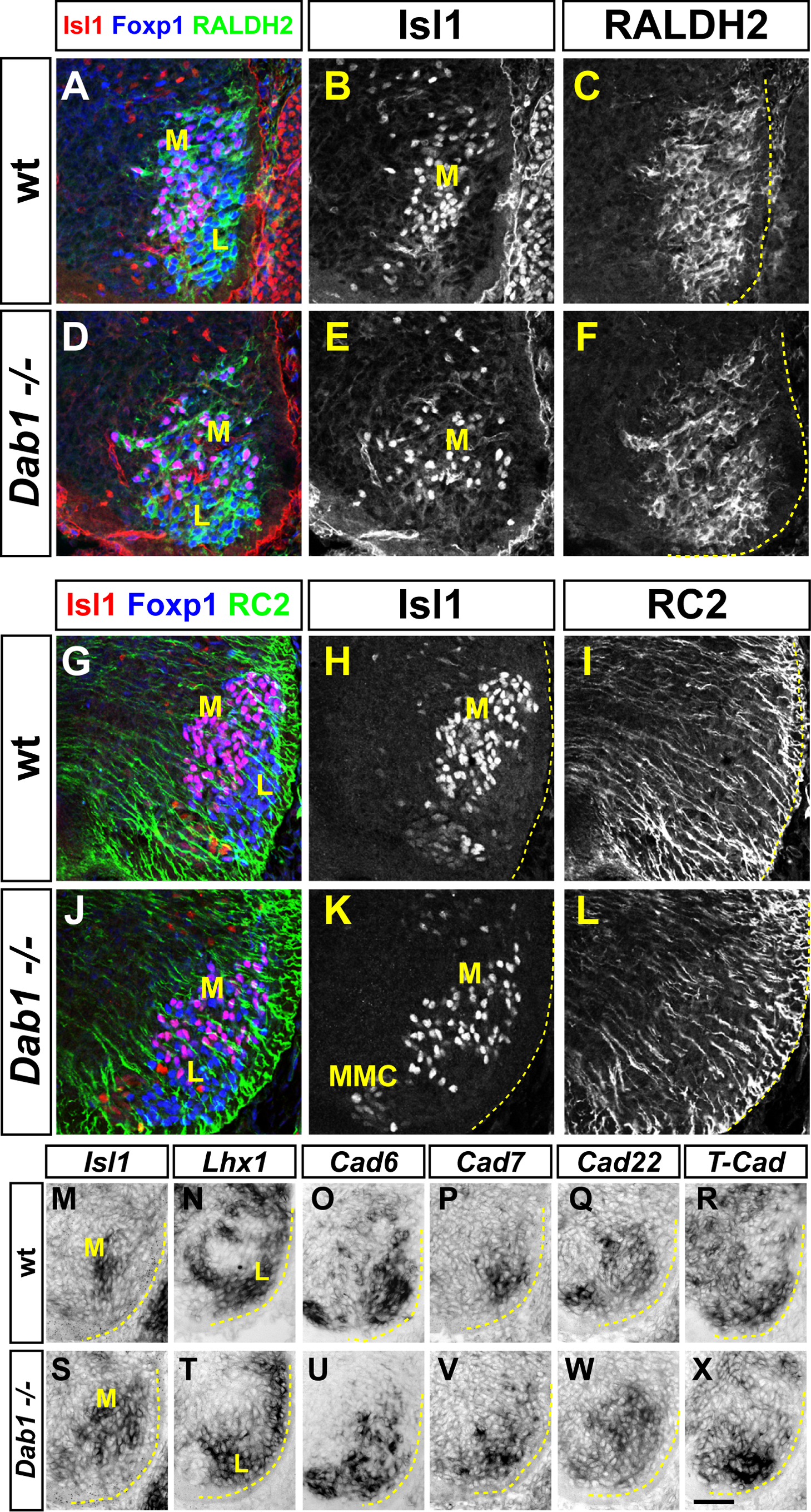

Supplement: Figure S2 — Expression of RALDH2, RC2, and Cadherins in Dab1 mutants. (A–L) Protein expression in lumbar spinal cord of e12.5 embryos. Foxp1 expression highlights LMC neurons; LMCm (M) neurons are Foxp1+ Isl1+, LMCl (L) neurons are Foxp1+ Isl1−. In Dab1 mutants, RALDH2 (A–F) and RC2 (G–L) distribution is unchanged when compared to control embryos. (M–X) Detection of cadherin mRNAs in consecutive sections of e12.5 lumbar spinal cord of Dab1 mutants (S–X) and control littermate (M–R). Isl1 (M, S) and Lhx1 (N, T) expression highlights LMCm (M) and LMCl (L) motor neurons, respectively. Scale bar: 50 µm (A–L) and 84 µm (M–X). (5.77 MB TIF) [file pbio.1000446.s002.tif]

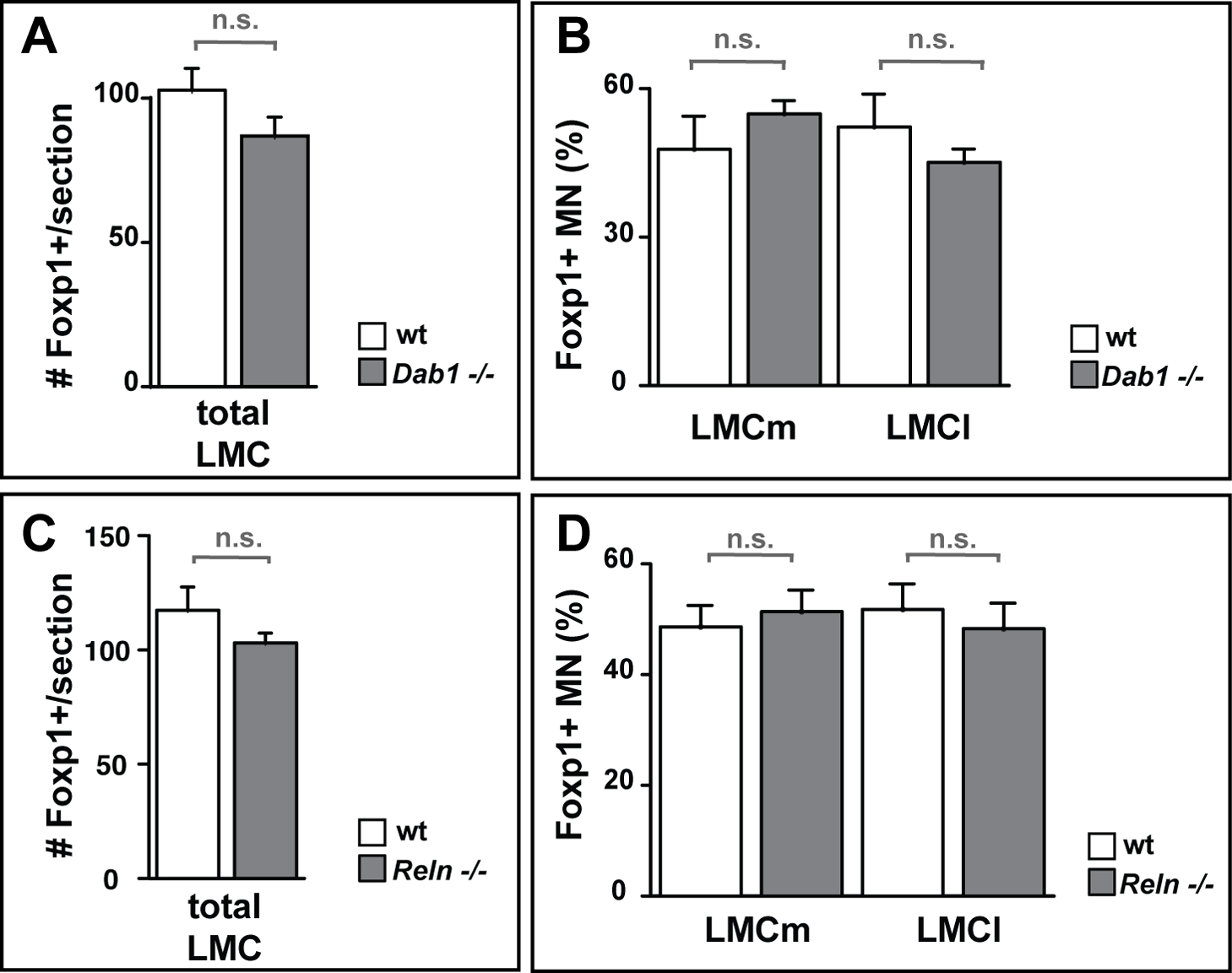

Supplement: Figure S3 — Quantification of motor neurons in Dab1 and Reln mutant embryos. (A, C) Quantification of LMC motor neuron numbers in e12.5 Dab1, Reln, and wild type (wt) littermate embryos expressed as the average number of total Foxp1+ LMC neurons per 12 µm section (# Foxp1+/section). LMC neuron numbers are not significantly different in mutants when compared to controls (p>0.17, Student's t test). (B, D) Quantification of LMCm (Isl1+ Foxp1+) and LMCl (Isl1− Foxp1+) motor neuron numbers in lumbar spinal cord of e12.5 Dab1 (B) and Reln (D) mouse embryos expressed as the percentage of all LMC motor neurons in 12 µm sections (Foxp1+ MN (%)). LMCm and LMCl neuron numbers are not significantly different in mutants when compared to controls (p>0.25 Student's unpaired t test). (0.32 MB TIF) [file pbio.1000446.s003.tif]

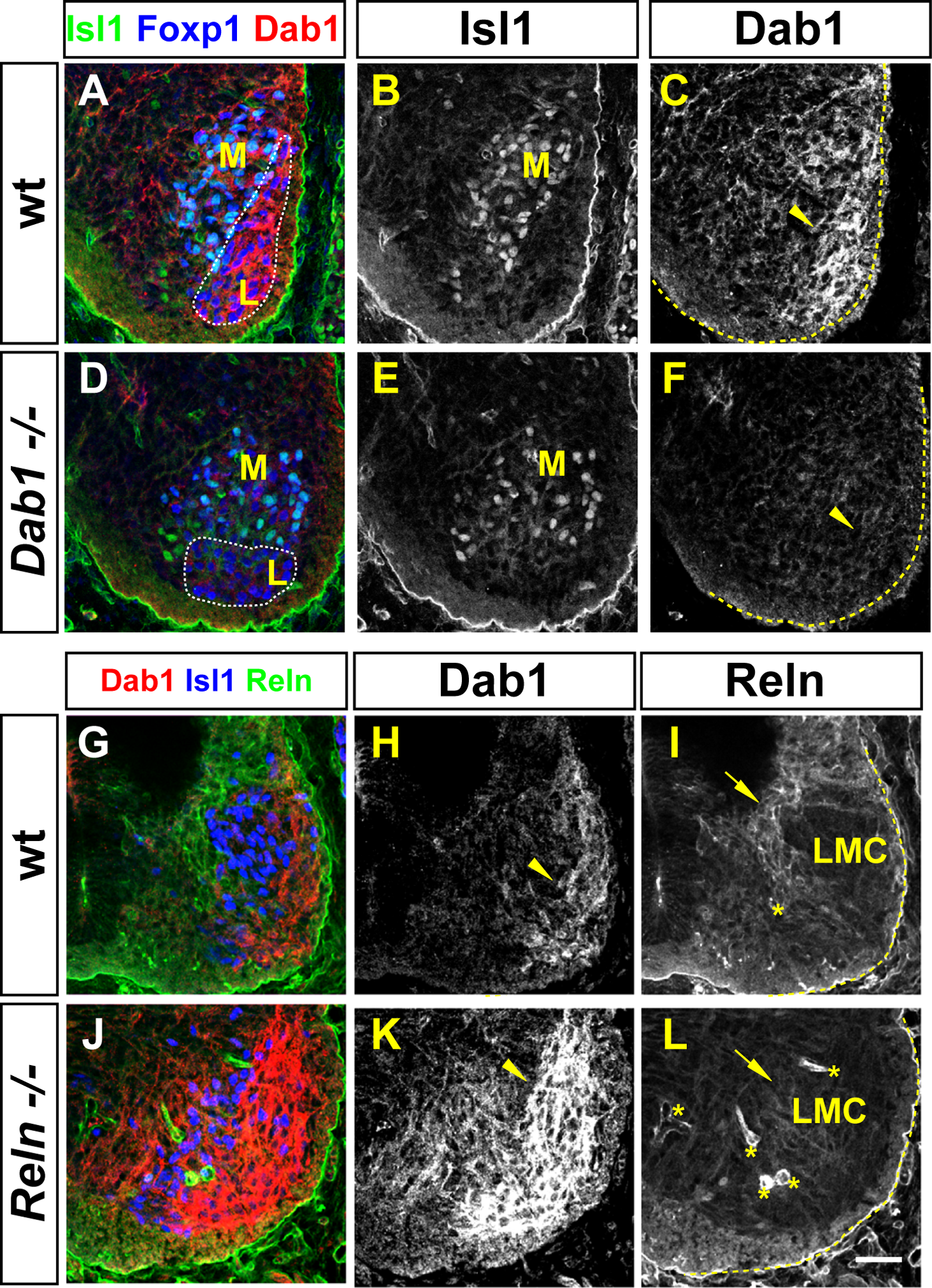

Supplement: Figure S4 — Characterisation of Dab1 and Reelin mutants. (A–F) Loss of Dab1 immunoreactivity (arrowheads) in the lumbar spinal cord of an e12.5 Dab1 mutant embryo (D–F) compared to a control littermate (A–C). LMCl (L) neurons are Foxp1+ Isl1− and LMCm (M) neurons are Foxp1+ Isl1+. (G–L) Loss of Reln protein immunoreactivity (arrows) in the lumbar spinal cord of a e12.5 Reln mutant embryo (J–L) compared to a control littermate (G, I). Dab1 protein levels are increased in the Reln mutant (J, K) relative to the control littermate (G, H) (arrowheads). Isl1 expression highlights LMCm neurons. Asterisks indicate blood vessels. Stippled lines outline the spinal gray or LMCl (L) neurons. Scale bar: 50 µm in all panels. (4.36 MB TIF) [file pbio.1000446.s004.tif]

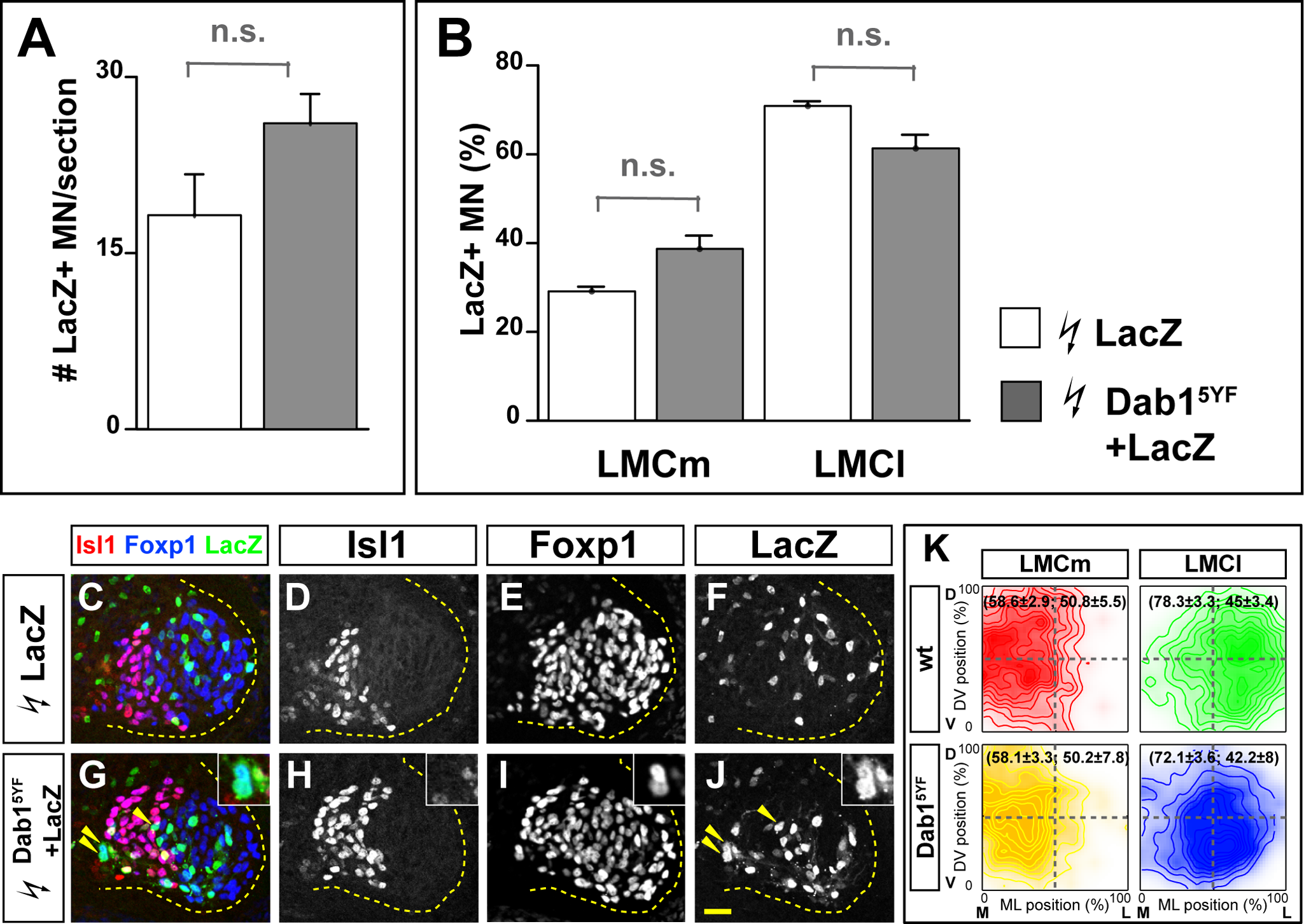

Supplement: Figure S5 — Cell autonomous requirement for Dab1 signaling in LMCl migration. (A) Quantification of LMC motor neurons electroporated with LacZ or Dab15YF and LacZ expression plasmids in chick HH St 29 embryos, expressed as the average number of the total LacZ+ LMC neurons per 12 µm section (# LacZ+ MN/section). The difference in numbers between control and experimental embryos is not significant (n.s.; p = 0.440, Student's unpaired t test; n = 7 embryos; N>100 neurons per embryo per experimental condition analysed). (B) Quantification of LMCm (Isl1+ Foxp1+) and LMCl (Isl1− Foxp1+) motor neurons in lumbar spinal cord of chick HH St29 embryos electroporated with LacZ or Dab15YF and LacZ expression plasmids, expressed as the percentage of total electroporated motor neurons [LacZ+ MN (%)]. The difference in numbers between control and experimental embryos is not significant (n.s.; p = 0.393, Student's unpaired t test; n = 7 embryos; N>100 neurons per embryo per experimental condition analysed). (C–J) Detection of LacZ, Foxp1, and Isl1 protein in LMCm (Foxp1+ Isl1+) and LMCl (Foxp1+ Isl1−) neurons in lumbar spinal cord of chick HH St 29 embryos electroporated with LacZ (C–F) or Dab15YF and LacZ expression plasmids (G–J). LMCl neurons expressing Dab15YF are frequently found in a zone ventromedial to LMCm neurons (arrowheads in J). Examples of electroporated LMCl neurons are indicated by arrowheads (G–J) and are shown at higher magnification (insets of G–J). (K) Density plots of mediolateral (ML) and dorsoventral (DV) position of electroporated LMCm and LMCl neurons expressed as percentage of LMC width and height. The position of LacZ-expressing LMCm neurons was not significantly different from Dab15YF-expressing LMCm embryos ((ML: 58.6%±2.9%; DV: 50.8%±5.5%) versus (ML: 58.1%±3.3%; DV: 50.2%±7.8%); p = 0.9235, Hotelling's T2 test). Dab15YF-expressing LMCl neurons were shifted significantly in a medial direction with respect to LacZ-expressing LMCl neurons ((ML: 72.1%±3.6%; DV: 42.2%± [file pbio.1000446.s005.tif]

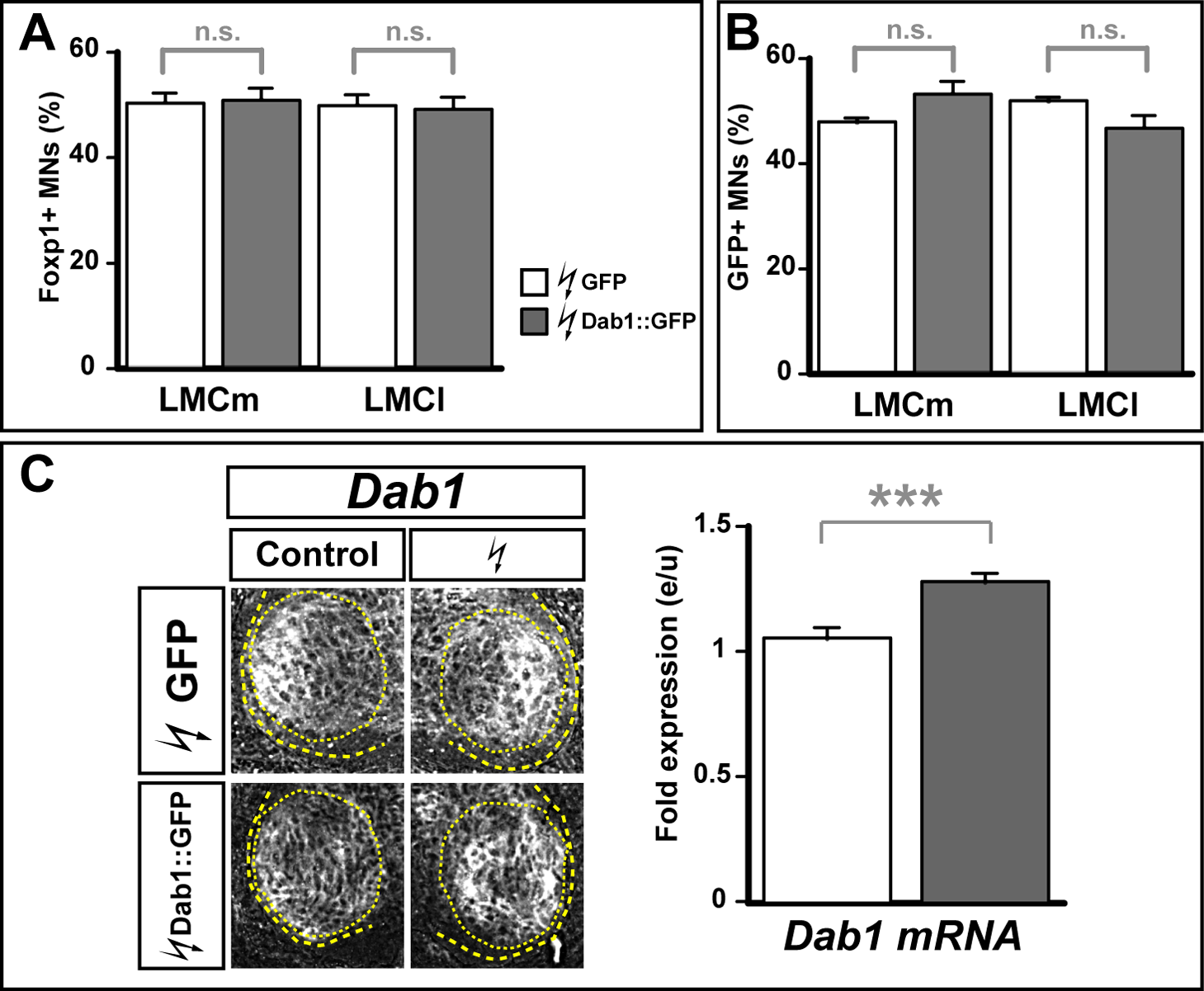

Supplement: Figure S6 — Quantification of Dab1 and LMC neuronal identity in embryos over-expressing Dab1. (A) Quantification of LMCm (Isl1+ Foxp1+) and LMCl (Foxp1+ Isl1−) neuron numbers in the lumbar spinal cord of chick HH St 29 embryos expressing GFP or Dab1::GFP, expressed as the percentage of total motor neurons (Foxp1+ MNs (%)). The difference between experimental and control embryos is not significant (p = 0.4324, Student's unpaired t test; n = 4 (GFP) and 5 (Dab1::GFP) embryos; N>60 neurons per embryo per experimental condition analysed). (B) Proportions of electroporated LMCm (Isl1+ Foxp1+ GFP+) and LMCl (Isl1− Foxp1+ GFP+) motor neurons in lumbar spinal cord of chick HH St29 embryos expressing GFP or Dab1::GFP (GFP+ MN (%)). The difference between experimental and control embryos is not significant (n.s.; p = 0.0510, Student's unpaired t test; n = 4 (GFP) and 5 (Dab1::GFP) embryos; N>90 neurons per embryo per experimental condition analysed). (C) In embryos electroporated with Dab1::GFP, Dab1 mRNA is upregulated on the electroporated side with respect to the unelectroporated side of the spinal cord. Quantification of Dab1 mRNA expression in GFP (white bar) or Dab1::GFP electroporated embryos (grey bar), normalized to the expression in the unelectroporated side of the spinal cord (electroporated/unelectroporated ratio: e/u). E/u ratio for Dab1 mRNA expression in GFP electroporated embryo is 1±0.04, while in Dab1::GFP electroporated embryos it is 1.3±0.03 (p<0.001; Student's t test; n = 3 embryos per experimental condition analysed). (0.59 MB TIF) [file pbio.1000446.s006.tif]

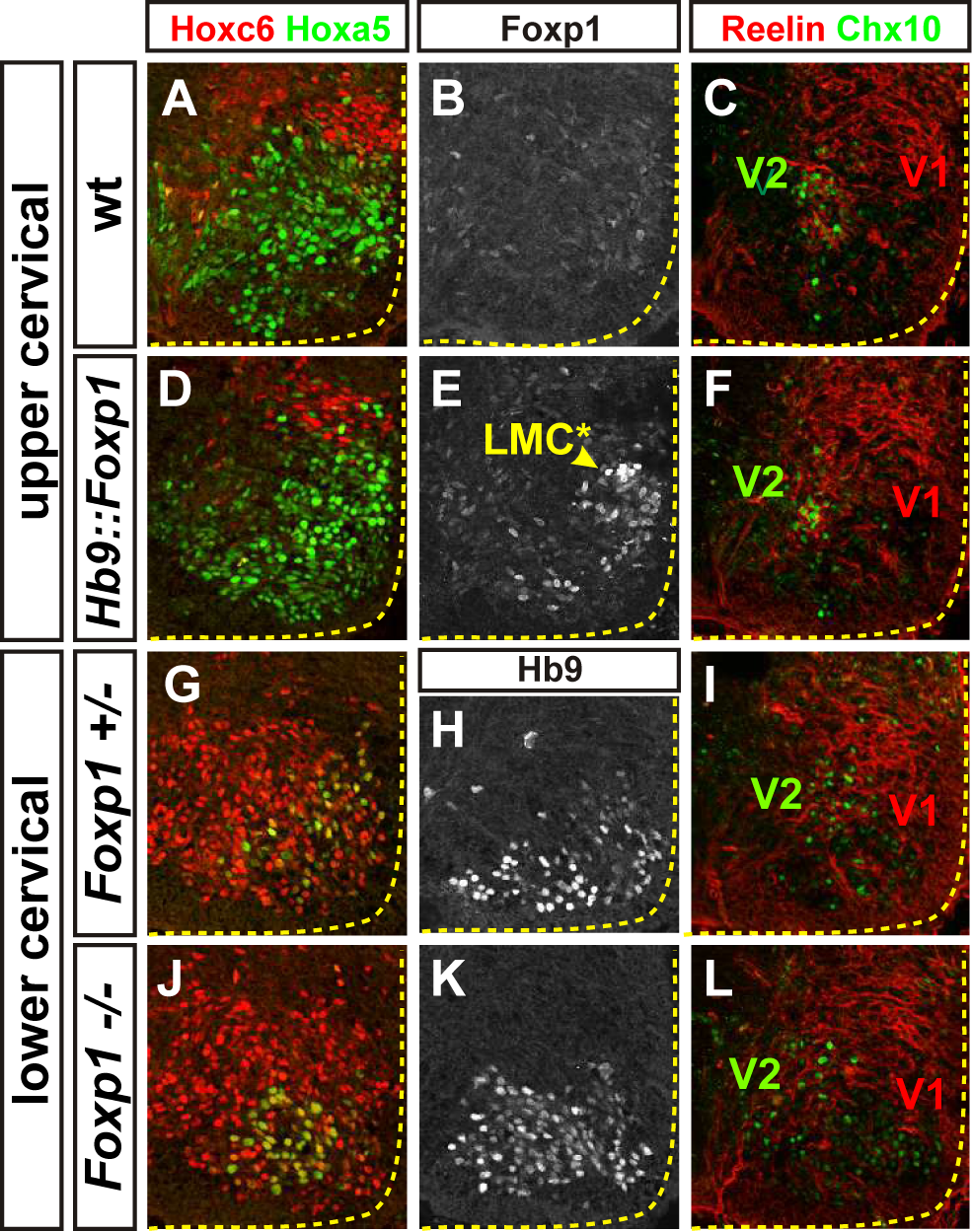

Supplement: Figure S7 — Normal Hoxa5, Hoxc6, Reelin, and Chx10 expression in Hb9::Foxp1 transgenics and Foxp1 mutants. (A, D, G, J) Hoxa5 and Hoxc6 protein detection in cervical spinal cord of Hb9::Foxp1 (D), Foxp1 mutant (J), and control littermate (A, G) e12.5 embryos. (B, E, H, K) Foxp1 and Hb9 detection in cervical spinal cord of Hb9::Foxp1 (E), Foxp1 mutant (K), and control littermate (B, H) e12.5 embryos. (C, F, I, L) Reelin and Chx10 detection in forelimbs of Hb9::Foxp1 (F), Foxp1 mutant (L), and control littermates (C, I) e12.5 embryos. LMC* indicates ectopic LMC in upper cervical sections of Hb9::Foxp1 embryos. (1.96 MB TIF) [file pbio.1000446.s007.tif]

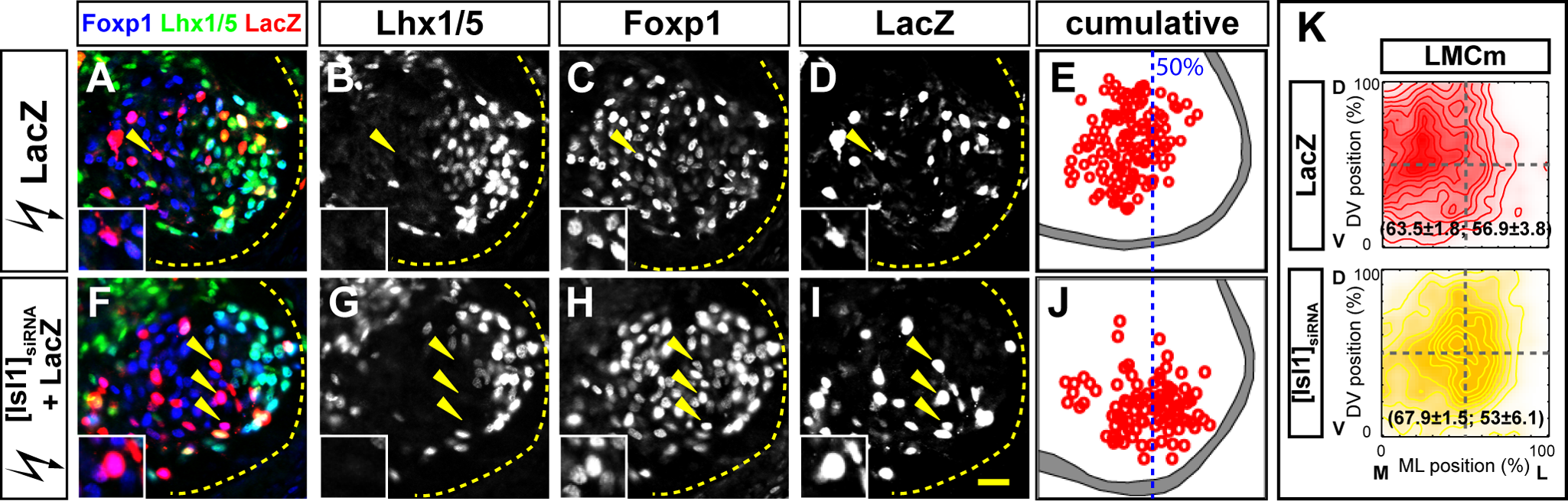

Supplement: Figure S8 — Isl1 is required for the specification of LMCm position. (A–D, F–I) Detection of LacZ, Foxp1, and Lhx1/5 protein in LMCm (Foxp1+ Lhx1/5−) and LMCl (Foxp1+ Lhx1/5+) neurons in lumbar spinal cord of chick HH St 29 embryos electroporated with LacZ (A–E) or [Isl1]siRNA and LacZ expression plasmids (F–J). Arrowheads point to electroporated LMCm neurons. Examples indicated by arrowheads are shown at higher magnification in insets of (A–D, F–I). (E, J) Superimposed ventral spinal cord location of electroporated LMCm (red) neurons in consecutive sections of representative embryos highlights the laterally shifted position of [Isl1]siRNA and LacZ expression plasmids electroporated LMCm neurons. The blue dashed line indicates the 50% ML value. (K) Density plots of mediolateral (ML) and dorsoventral (DV) positions of electroporated LMCm and LMCl neurons expressed as percentage of LMC width and height. The mean position of [Isl1]siRNA-electroporated LMCm neurons (ML: 67.9%±1.5%; DV: 53%±6%) was significantly shifted laterally with respect to the mean position of LacZ-expressing LMCm neurons ((ML: 63.5%±1.8%; DV: 56.9%±3.8%); p = 0.0473, Hotelling's T2 test). Number of embryos analysed per experimental condition: 4 (LacZ) and 5 (LacZ+ [Isl1]siRNA); number of neurons included in the analysis: >60 per embryo per experimental condition. Dashed lines divide LMC in four equal quadrants. All values are expressed as mean ± s.d. Yellow stippled lines outline the spinal gray matter. Scale bar: 23 µm in all panels. (1.37 MB TIF) [file pbio.1000446.s008.tif]

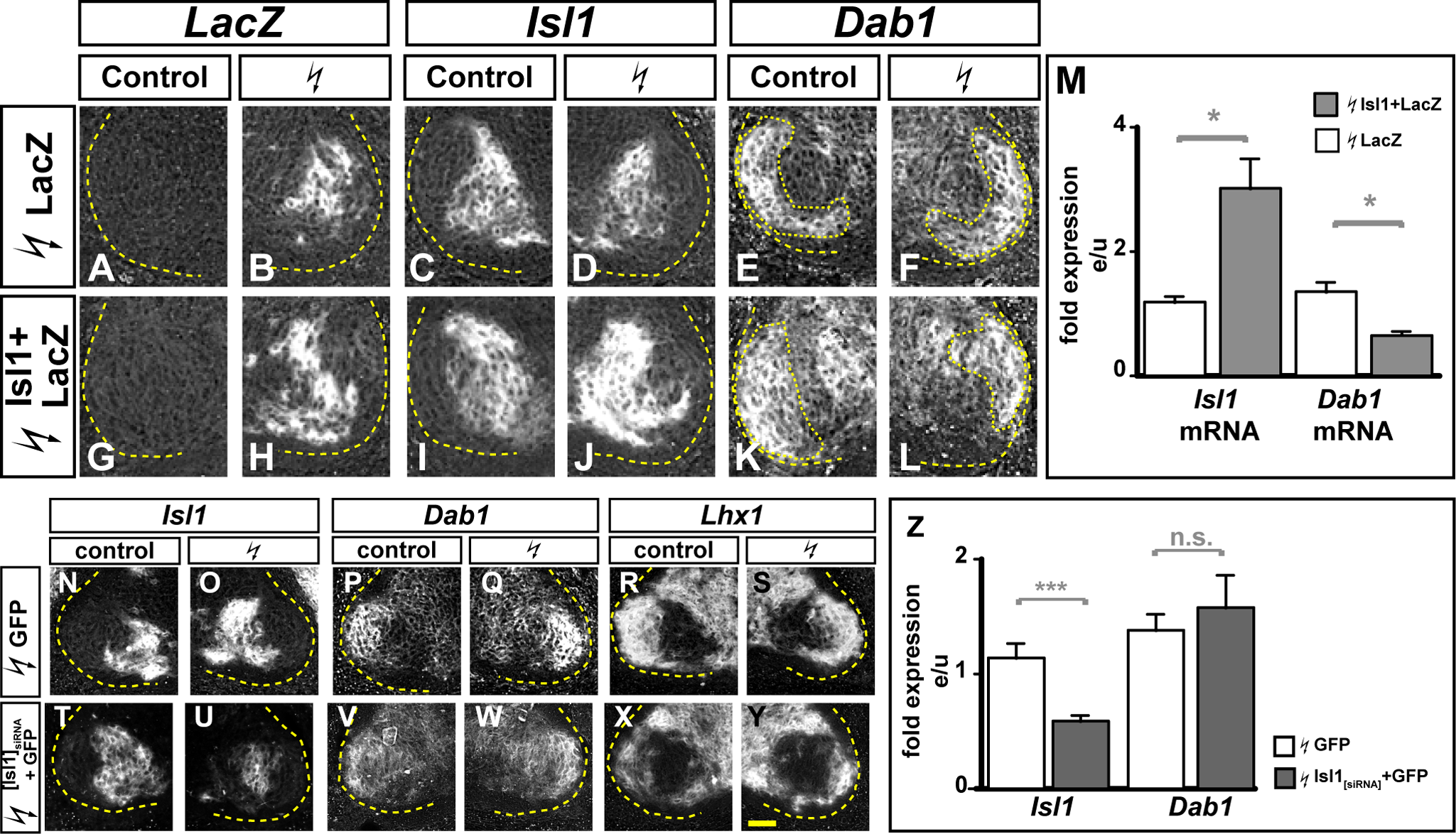

Supplement: Figure S9 — Isl1 is sufficient to regulate Dab1 mRNA expression. (A–L, N–Y) LacZ, Isl1, Lhx1, and Dab1 mRNA detection in chick HH St 28–30 embryos electroporated with LacZ (A–F), Isl1 and LacZ expression plasmids (G–L), GFP (N–S), or [Isl1]siRNA and GFP expression plasmid (T–Y). In embryos electroporated with Isl1, Dab1 is downregulated on the electroporated side (L) with respect to the unelectroporated side of the spinal cord (K). No significant effect on Dab1 expression was observed in LacZ electroporated embryos (E, F). (M) Quantification of Isl1 and Dab1 mRNA expression in LacZ (white bars) or Isl1 electroporated embryos (grey bars), with values normalized to expression in the unelectroporated side of the spinal cord (electroporated/unelectroporated ratio: e/u). E/u ratio for Isl1 mRNA expression in LacZ electroporated embryo is 1.2±0.1, while in Isl1 electroporated embryos it is 3±0.5 (p = 0.002; Student's t test). Dab1 mRNA expression e/u ratio in Isl1 electroporated embryos (0.7±0.1) is significantly different from that in LacZ electroporated embryos (1.4±0.1; p<0.001, Student's t test). (Z) Quantification of numbers of Isl1 or Foxp1 expressing neurons in GFP- (white bars) or [Isl1]siRNA-electroporated embryos (grey bars), expressed as the ratio between electroporated/unelectroporated spinal cord sides (e/u). E/u value for Isl1+ Foxp1+ neurons in GFP electroporated embryos is 1±0.03, and in [Isl1]siRNA electroporated embryos it is 0.8±0.05 (p<0.001; Student's t test). Foxp1+ neurons in [Isl1]siRNA electroporated embryos (0.95±0.0.3) is not significantly different from GFP electroporated embryos (1±0.0.2; p<0.075, Student's t test). All values are expressed as mean ± s.e.m. Fine stippled lines highlight Dab1high area, heavy stippled lines outline the spinal gray. Number of embryos analysed per experimental condition: 3. Scale bar: 59 µm (A–L) and 46 µm (N–Y). (1.82 MB TIF) [file pbio.1000446.s009.tif]

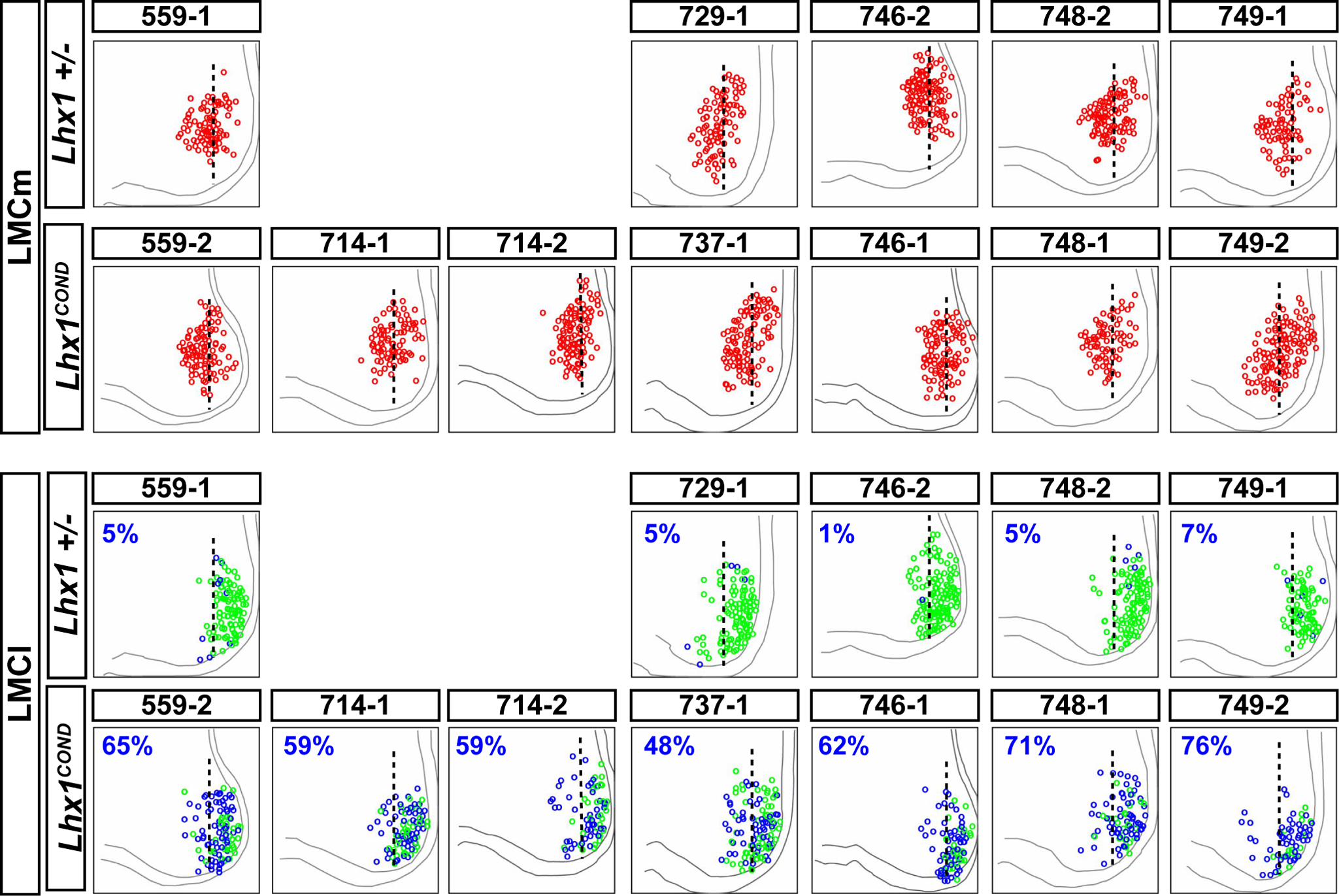

Supplement: Figure S10 — Variability of LMCl neuron location in Lhx1 mutants. Superimposed spinal cord position of LMCm (red), LMCl (green), and LMCl* (blue) neurons in consecutive sections of analyzed embryos highlights altered position of LMCl* neurons. Blue numbers indicate the fractions of LMCl* neurons expressed as percentage of Isl1− Foxp1+ motor neurons and the dashed line represents the 50% ML value. (1.59 MB TIF) [file pbio.1000446.s010.tif]
