# Supplementary material for: Foxp1 and Lhx1 Coordinate Motor Neuron Migration with Axon Trajectory Choice by Gating Reelin Signalling
Source: PLoS Biol. 2010 Aug 10;8(8):e1000446. doi: 10.1371/journal.pbio.1000446 (PMC2919418; doi:10.1371/journal.pbio.1000446)
Supplement: Table S1 — Antibodies used in this study. (A) Primary antibodies, (B) fluorochrome-conjugated secondary antibodies. DSHB: Developmental Studies Hybridoma Bank, developed under the auspices of the NICHD and maintained by The University of Iowa, Department of Biological Sciences, Iowa City, IA 52242. (0.09 MB DOC) [file pbio.1000446.s011.doc]

1. Primary antibodies

| **Antigen** | **Species source** | **Dilution** | **Source/Reference** |
| --- | --- | --- | --- |
| Chicken NF  related protein | Mouse (3A10) | 1:100 | DSHB |
| Chx10 | Rabbit | 1:8000 | [1] |
| Dab1 | Rabbit | 1:1000 | Chemicon |
| Foxp1 | Rabbit | 1:2000 | Abcam |
|  | Guinea pig | 1:16000 | [2] |
| GFP | Sheep | 1:1000 | Biogenesis |
| Hb9 | Guinea pig | 1:20000 | [3] |
|  | Mouse (81.5C10) | 1:100 | DSHB |
| Hoxa5 | Rabbit | 1:5000 | [4] |
| Hoxc6 | Goat | 1:100 | Abcam |
| Isl1 | Mouse (39.3F7) | 1:100 | DSHB [5] |
| Isl1/2 | Rabbit (K4) | 1:20 000 | [5] |
| LacZ | Rabbit | 1:4000 | Rockland |
| Lhx1/5 | Mouse (4F2) | 1:100 | DSHB [5] |
|  | Rabbit | 1:3000 | [1] |
| Lhx3 | Mouse (67.4E12) | 1:100 | DSHB |
| Mouse NF | Mouse (2H3) | 1:100 | DSHB |
| Myc | Mouse (9E10) | 1:100 | DSHB |
| Pea3 | Rabbit | 1:5000 | [6] |
| P-Smad1 | Rabbit | 1:1000 | E. Laufer |
|  | Guinea pig | 1:500 | E. Laufer |
| RALDH2 | Rabbit | 1:4000 | [7] |
| RC2 | Mouse | 1:100 | DSHB |
| Reln | Mouse (G10) | 1:1000 | Calbiochem |
|  | Mouse (142) | 1:1000 | Abcam |
| VLDLR | Goat | 1:1000 | R&D System |

1. Fluorochrome-conjugated secondary antibodies

| Mouse IgM | Goat Alexa-488 | 1:1000 | Invitrogen |
| --- | --- | --- | --- |
| Mouse IgG  Rabbit IgG  Sheep IgG  Goat IgG | Donkey Alexa-488 | 1:1000 | Invitrogen |
| Mouse IgG  Rabbit IgG | Donkey Cy3 | 1:1000 | Jackson Immunoresearch Labs |
| Guinea pig IgG  Rabbit IgG | Donkey Cy5 | 1:500 | Jackson Immunoresearch Labs |

**Supplemental Table 1 References**

1. Ericson J, Rashbass P, Schedl A, Brenner-Morton S, Kawakami A, et al. (1997) Pax6 controls progenitor cell identity and neuronal fate in response to graded Shh signaling. Cell 90: 169-180.

2. Rousso DL, Gaber ZB, Wellik D, Morrisey EE, Novitch BG (2008) Coordinated actions of the forkhead protein Foxp1 and Hox proteins in the columnar organization of spinal motor neurons. Neuron 59: 226-240.

3. Thaler J, Harrison K, Sharma K, Lettieri K, Kehrl J, et al. (1999) Active suppression of interneuron programs within developing motor neurons revealed by analysis of homeodomain factor HB9. Neuron 23: 675-687.

4. Dasen JS, Tice BC, Brenner-Morton S, Jessell TM (2005) A Hox regulatory network establishes motor neuron pool identity and target-muscle connectivity. Cell 123: 477-491.

5. Tsuchida T, Ensini M, Morton SB, Baldassare M, Edlund T, et al. (1994) Topographic organization of embryonic motor neurons defined by expression of LIM homeobox genes. Cell 79: 957-970.

6. Livet J, Sigrist M, Stroebel S, De Paola V, Price SR, et al. (2002) ETS gene Pea3 controls the central position and terminal arborization of specific motor neuron pools. Neuron 35: 877-892.

7. Sockanathan S, Jessell TM (1998) Motor neuron-derived retinoid signaling specifies the subtype identity of spinal motor neurons. Cell 94: 503-514.
