# Supplementary material for: Foxp1 and Lhx1 Coordinate Motor Neuron Migration with Axon Trajectory Choice by Gating Reelin Signalling
Source: PLoS Biol. 2010 Aug 10;8(8):e1000446. doi: 10.1371/journal.pbio.1000446 (PMC2919418; doi:10.1371/journal.pbio.1000446)
Supplement: Table S2 — Position of LMC neurons in Dab1 and Reln mutants. n: number of embryos analysed; N: total numbers of neurons counted; p values for position of mutant versus littermate wild type neurons are from the randomized Hotelling's T2 test under unequal variances. a Values are ± standard deviation of the mean. (0.04 MB DOC) [file pbio.1000446.s012.doc]

|  | **LMCm** | | | | | | **LMCl** | | | | | |
| --- | --- | --- | --- | --- | --- | --- | --- | --- | --- | --- | --- | --- |
| **Genotype** | **ML[%]^a^** | **DV[%]^a^** | **n** | **N** | **N/embryo** | **p** | **ML[%]^a^** | **DV[%]^a^** | **n** | **N** | **N/embryo** | **p** |
| *Dab1*+/+ | 75.1±2 | 66.7±4.3 | 4 | 759 | 189.8±73.7 |  | 78.9±1.6 | 39.0±4 | 4 | 907 | 226.8±79.8 |  |
| *Dab1*-/- | 74.9±1.5 | 62.6±2.9 | 6 | 1089 | 181.5±55.6 | 0.2925 | 73.0±1.1 | 32.6±3.4 | 6 | 809 | 134.8±34.5 | 0.0035 |
| *Reln*+/+ | 73.9±2.2 | 60±4 | 4 | 781 | 195.3±61.9 |  | 80.4±1.9 | 40.9±4.2 | 4 | 790 | 197.5±50.8 |  |
| *Reln*-/- | 74.1±2 | 61.5±7.5 | 4 | 907 | 226.8±65.5 | 0.9024 | 74.8±2 | 35.3±3.7 | 4 | 859 | 214.8±86.8 | 0.0473 |
