# Supplementary material for: Foxp1 and Lhx1 Coordinate Motor Neuron Migration with Axon Trajectory Choice by Gating Reelin Signalling
Source: PLoS Biol. 2010 Aug 10;8(8):e1000446. doi: 10.1371/journal.pbio.1000446 (PMC2919418; doi:10.1371/journal.pbio.1000446)
Supplement: Table S3 — Position of LMC neurons in electroporated chick embryos. n: number of embryos analysed; N: total numbers of neurons counted; p values for position of experimental versus control neurons are from randomized Hotelling's T2 test under unequal variances. a Values are ± standard deviation of the mean; b only electroporated, LacZ+, or GFP+ LMC neurons were included in the analysis; c only electroporated LacZ+ Lhx1/2− LMC neurons were included in the analysis. (0.06 MB DOC) [file pbio.1000446.s013.doc]

|  | **LMCm** | | | | | | **LMCl** | | | | | | |
| --- | --- | --- | --- | --- | --- | --- | --- | --- | --- | --- | --- | --- | --- |
| **Plasmids electroporated** | **ML[%]^a^** | **DV[%]^a^** | **n** | **N** | **N/embryo** | **p** | **ML[%]^a^** | **DV[%]^a^** | **n** | **N** | **N/embryo** | **p** |  |
| LacZ^b^ | 58.6±2.9 | 50.8±5.5 | 7 | 299 | 42.7±21.9 |  | 78.3±3.3 | 45±3.4 | 7 | 522 | 74.6±26.6 |  |  |
| Dab1^5YF^ +LacZ^b^ | 58.1±3.3 | 50.2±7.8 | 7 | 378 | 54±20.1 | 0.9235 | 72.1±3.6 | 42.2±8 | 7 | 595 | 85±29.2 | 0.0233 |  |
| GFP^b^ | 67.4±2.2 | 58.9±4.2 | 4 | 101 | 25.3±18 |  | 83.5±3.1 | 52.2±4.5 | 4 | 154 | 38.5±25.9 |  |  |
| Dab1::GFP^b^ | 70.2±2.4 | 49.1±3.6 | 5 | 178 | 35.6±6.3 | 0.0165 | 84.2±1.3 | 53±6 | 5 | 168 | 33.6±4 | 0.9019 |  |
| LacZ^c^ | 63.5±1.8 | 56.9±3.8 | 4 | 333 | 70.7±8 |  | n.a. | n.a. | n.a. | n.a. | n.a. | n.a. |  |
| [Isl1]_siRNA_+ LacZ^c^ | 67.9±1.5 | 53±6.1 | 3 | 322 | 68.5±8 | 0.0473 | n.a. | n.a. | n.a. | n.a. | n.a. | n.a. |  |
