# Supplementary material for: Foxp1 and Lhx1 Coordinate Motor Neuron Migration with Axon Trajectory Choice by Gating Reelin Signalling
Source: PLoS Biol. 2010 Aug 10;8(8):e1000446. doi: 10.1371/journal.pbio.1000446 (PMC2919418; doi:10.1371/journal.pbio.1000446)
Supplement: Table S4 — Position of LMC neurons in Lhx1 mutants. LMCm (A), LMCl and LMCl* (B) position analysis. n: number of embryos analyzed; N: total numbers of neurons counted; p values for position versus littermate Lhx1 +/−, Randomization Hotellings T2 test under unequal variances; p′ values for position LMCl* versus LMCl same genotype, Randomization Hotellings T2 test under unequal variances; p″ values for position LMCl* versus LMCl littermate Lhx1+/ −, Randomization Hotellings T2 test under unequal variances. a Values are ± standard deviation of the mean. (0.05 MB DOC) [file pbio.1000446.s014.doc]

A. LMCm position analysis

|  | **LMCm** | | | | | |
| --- | --- | --- | --- | --- | --- | --- |
| **Genotype** | **ML[%]^a^** | **DV[%]^a^** | **n** | **N** | **N/embryo** | **p** |
| *Lhx1 +/-* | 73.4±3.7 | 62.9±3 | 5 | 872 | 174.4±48.1 |  |
| *Lhx1 -/-* | 77.7±2.8 | 63.9±3.2 | 7 | 951 | 135.9±50.9 | 0.1613 |

B. LMCl and LMCl* position analysis

|  | **LMCl** | | | | | | **LMCl*** | | | | | | | |
| --- | --- | --- | --- | --- | --- | --- | --- | --- | --- | --- | --- | --- | --- | --- |
| **Genotype** | **ML[%]^a^** | **DV[%]^a^** | **n** | **N** | **N/embryo** | **p** | **ML[%]^a^** | **DV[%]^a^** | **n** | **N** | **N/embryo** | **p** | **p’** | **p’’** |
| *Lhx1 +/-* | 80.9±1.4 | 40.2±2.4 | 5 | 743 | 148.6±34.3 |  | 77.1±5.4 | 62.3±16.6 | 5 | 37 | 7.4±3.8 |  | 0.0157 |  |
| *Lhx1 -/-* | 82.1±2.2 | 35.4±5.6 | 7 | 266 | 38±15.9 | 0.175 | 80.9±1.8 | 39.8±6.4 | 7 | 430 | 61.4±17.4 | 0.0087 | 0.2825 | 0.9886 |
