# Supplementary material for: Foxp1 and Lhx1 Coordinate Motor Neuron Migration with Axon Trajectory Choice by Gating Reelin Signalling
Source: PLoS Biol. 2010 Aug 10;8(8):e1000446. doi: 10.1371/journal.pbio.1000446 (PMC2919418; doi:10.1371/journal.pbio.1000446)
Supplement: Text S1 — Supplemental data and supplemental materials and methods. (0.05 MB DOC) [file pbio.1000446.s015.doc]

**Text S1**

**Isl1 is required for the localisation of LMC neurons**

We asked whether Isl1 not only was sufficient [1], but also

required to determine LMC neuron position. We therefore electroporated *in ovo* siRNA

against Isl1 ([Isl1]siRNA ) together with a LacZ expression plasmid [2] in

the lumbar spinal cord of HH St 17/18 and monitored the position of LacZ^+^ LMCm

neurons at HH St29. At the stages relevant to LMC neuron migration, the total number of

LMC neurons and LMCl subtype specification as assessed by Hb9, FoxP1 and Lhx1/5

expression was unaffected [2] (data not shown), however electroporation

of [Isl1]siRNA resulted in decreased number of neurons expressing Isl1 when compared to the unelectroporated side of the spinal cord or LacZ control animals (Fig. S9; data not

shown). In all embryos analysed, LMCm neurons electroporated with [Isl1]siRNA (defined as FoxP1^+^Lhx1/5^-^ LacZ^+^) were observed in a more lateral position (Fig. S8; (ML: 68%; DV: 53%)) compared to LMCm neurons electroporated with LacZ ((ML: 63%; DV: 57%), p=0.0473, Hotelling’s T^2^ test; Fig. S8; Table S3). These data demonstrate that Isl1 is required for the correct medial position of LMCm neurons.

We next analyzed whether lack of Isl1 expression affects Dab1 transcription. [Isl1]siRNA

together with GFP expression plasmid [2] or GFP alone as control were

introduced by *in ovo* elctroproation in the lumbar spinal cord of HH St 17/18 and *Dab1*

mRNA levels were examined in the area corresponding to LMCm (defined as Lhx1^-^) of

the electroporated side of the spinal cord respect to the unelectroporated side at HH St 29

[1]. Quantification of *Dab1* mRNA indicate no significant

difference in embryos electroporated with [Isl1]siRNA respect to control embryos (Fig.

S9; e/u values: 1.6 vs. 1.38 in control; p=0.496 Student’s T test). These data indicate

that Isl1 is required for the medial position of LMCm neurons, but not to modulate Dab1

mRNA expression levels.

**Supplemental Materials and Methods**

**Motor neuron position quantification**

Distance (D) and angle (α) from the ventral edge of the ventricular zone to the motor

neuron soma (D_MN_; α_MN_) and the most lateral neuron of the LMC or, when indicated, the

most pial surface of the ventral spinal cord (D_SC_; α_SC_) were measured using ImageJ

(NIH) image analysis software. The distance between the most ventrally (MNV) and

dorsally (MND) located motor neurons somas defined LMC height

[LMC_height_=(D_MND_*sinα_MND_) -(D_MNV_*sinα_MNV_)]. ML position and DV position were

defined as (D_MN_ * cosα_MN_)/(D_SC_ * cosα_SC_) and [(D_MN_ *sinα_MN_)−(D_MNV_*sinα_MNV_)]/ LMC_height_ respectively and the location of the LMC was defined in control embryos as that containing 95% of Foxp1^+^ neurons. (ML; DV) measurement of neurons located in the LMC were plotted using Matlab software (Mathworks, Canada).

**Expression plasmids generation**

Tyrosines Y185, Y198, Y200, Y220, Y232 were mutated to phenylalanine using standard PCR based methodology and the following primers: (Y185F-5)

GTGTGAACAGGCGGTATTCCAGACAATTTTGGAAGAAG, (Y198/200F-5)

GTAGAAGACCCTGTATTCCAGTTCATTGTGTTTGAGGCTG, (Y220F-5)

ACAGAAGAAAACATTTTTCAGGTTCCTACCAGC, (Y232F-5)

AAGAAGGAAGGTGTTTTTGATGTGCCAAAAAGTC. The resulting Dab15YF gene

was fused at the C-terminus downstream of a CMV-LoxP-STOP-LoxP cassette in

pCAGGS [3].

The Dab15YF expression plasmid was co-electroporated with a pCDNA3.1 plasmid

(Invitrogen, Israel) expressing Cre under Hb9 promoter (Hb9::Cre [4,5]) at a 1:1 DNA mass ratio and LacZ expression plasmid at 4:1

DNA mass ratio [1].

**Supplemental References**

1. Kania A, Jessell TM (2003) Topographic motor projections in the limb imposed by LIM homeodomain protein regulation of ephrin-A:EphA interactions. Neuron 38: 581-596.

2. Luria V, Krawchuk D, Jessell TM, Laufer E, Kania A (2008) Specification of motor axon trajectory by ephrin-B:EphB signaling: symmetrical control of axonal patterning in the developing limb. Neuron 60: 1039-1053.

3. Zisman S, Marom K, Avraham O, Rinsky-Halivni L, Gai U, et al. (2007) Proteolysis and membrane capture of F-spondin generates combinatorial guidance cues from a single molecule. J Cell Biol 178: 1237-1249.

4. Lee SK, Jurata LW, Funahashi J, Ruiz EC, Pfaff SL (2004) Analysis of embryonic motoneuron gene regulation: derepression of general activators function in concert with enhancer factors. Development 131: 3295-3306.

5. Wichterle H, Lieberam I, Porter JA, Jessell TM (2002) Directed differentiation of embryonic stem cells into motor neurons. Cell 110: 385-397.
